# Optically Pure Calixarenyl Phosphine via Stereospecific Alkylation on Evans’ Oxazolidinone Moiety

**DOI:** 10.3390/molecules29051156

**Published:** 2024-03-05

**Authors:** Claude Bauder, David Sémeril

**Affiliations:** Synthèse Organométallique et Catalyse, UMR-CNRS 7177 Institut de Chimie de Strasbourg, Strasbourg University, 67008 Strasbourg, France

**Keywords:** Evans’ oxazolidinone, calix[4]arene, chirality, phosphine, ruthenium, asymmetric reduction

## Abstract

A convenient protocol for the synthesis of 25,26,27-tribenzoyl-28-[((*S*)-1-diphenylphos- phanyl-propan-2-yl)oxy]-calix[4]arene via stereospecific methylation on Evans’ oxazolidinone moiety was reported. According to the ^13^C NMR analysis of this phosphine, the calix[4]arene skeleton adopted a 1,3-alternate conformation. The latter conformation of the macrocycle and the (*S*)-chirality of the carbon atom bearing the methyl substituent were confirmed by a single-crystal X-ray diffraction study. After coordination of the phosphinated ligand to the dimeric [RuCl_2_(*p*-cymene)]_2_ organometallic precursor, the resulting arene–ruthenium complex was tested in the asymmetric reduction of acetophenone and alcohol was obtained with modest enantiomeric excess.

## 1. Introduction

Calix[4]arenes are macrocyclic compounds formed by condensation of four molecules of phenol and four molecules of formaldehyde, which, after adequate functionalization, could be used as building blocks for applications in host–guest chemistry, coordination chemistry, homogeneous catalysis, and materials or medicinal sciences [1,2,3,4,5,6,7,8,9,10,11,12].

**Figure 1:**
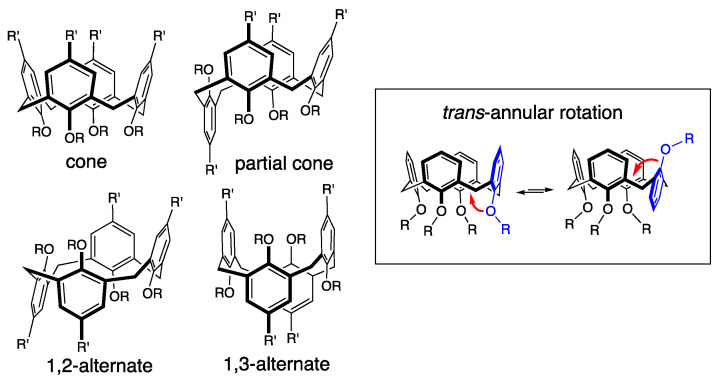
Conformational isomeric structures of calix[4]arene.

Furthermore, calix[4]arenes are dynamic molecules that can exist under the following four conformational isomeric structures: cone, partial cone, 1,2-alternate, and 1,3-alternate (Figure 1). Ring inversion occurs through “oxygen-through-the-annulus rotation” [13] and it is well-established that the introduction of propyl substituents at the phenolic positions is sufficient to block this rotation and lock the conformation of calix[4]arenes [14].

**Figure 2:**
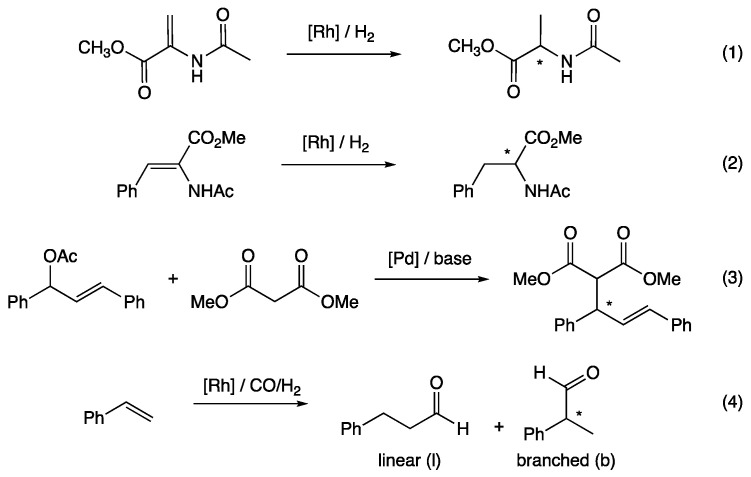
Examples of reported asymmetric reactions involving phosphinated calix[4]arenes as optically active ligands.

With appropriate functionalization, generic calix[4]arene provides a useful preorganization platform for the preparation of optically active phosphinated ligands. As examples, these can be used in the asymmetric reduction of olefins or in the asymmetric formation of carbon–carbon bonds (Figure 2).

**Figure 3:**
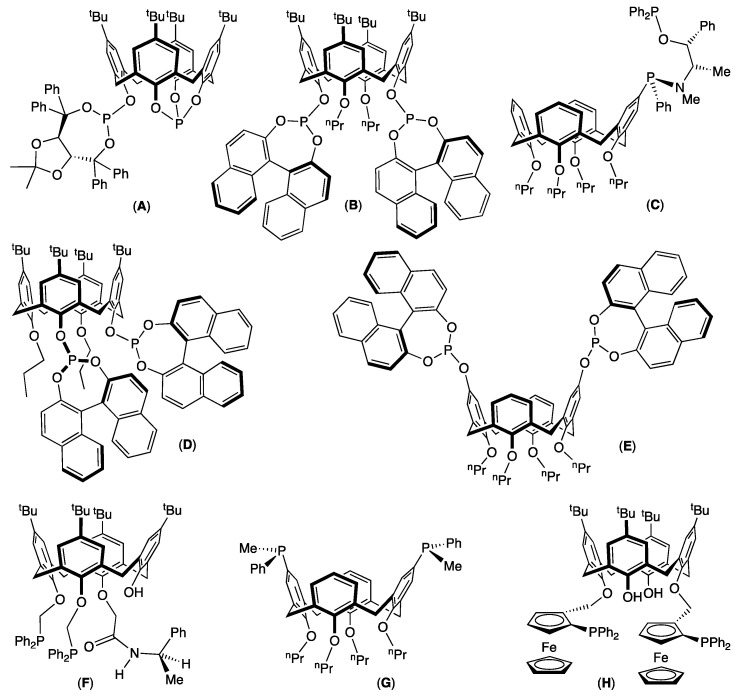
Selection of optically active phosphinated ligands (**A**–**H**) based on a calix[4]arene skeleton.

For instance, for the asymmetric rhodium-catalyzed hydrogenation of methyl-(*Z*)-2-(acetamido)acrylate (Equation (1) in Figure 2), van Leeuwen and co-workers reported the use of calixarenyl diphosphite **A** (Figure 3) based on the (*R*,*R*)-2,2-dimethyl-α,α,α’,α’-tetraphenyldioxolane-4,5-dimethanol moiety. The catalytic system was generated in situ from the ligand and [Rh(nbd)_2_]BF_4_ (nbd = bicyclo[2.2.1]hepta-2,5-diene) as an organometallic precursor (1 mol%) in dichloromethane. After 20 h at room temperature under 5 bar of hydrogen, the (*R*)-alanine derivative was quantitatively obtained with an enantiomeric excess (ee) of 94% [15]. A slightly higher enantioselectivity (ee = 98%) was obtained by Sandoval and co-workers using [Rh(cod)_2_]BF_4_ (1 mol%; cod = 1,5-cyclooctadiene) and the non-conformationally flexible calixarenyl diphosphite **B** (Figure 3) as the catalytic system. The run was performed in dichloromethane under 5 bar of hydrogen at 30 °C for 11 h [16].

Using the ephedrine methodology, Harvey, Jugé, and co-workers synthetized in two steps aminophosphine–phosphinite **C** (Figure 3). Catalytic hydrogenation was carried out using the well-defined [Rh(cod)(**C**)]BF_4_ complex (3 mol%) in benzene under 15 bar of hydrogen. After 16 h, full reduction of the substrate was observed and (*R*)-methyl 2-acetamidopropionate was formed with a modest enantioselectivity (ee = 34%). Repeating the run with (*Z*)-*N*-acetyl-dehydro-phenylalanine methyl ester as the substrate (Equation (2) in Figure 2), under only 1 bar of hydrogen, enantioselectivity of the reduced (*R*)-methyl 2-acetamido-3-phenylpropionate reached the value of 98% [17].

In the case of asymmetric rhodium-catalyzed hydrogenation of methyl ester (*Z*)-*N*-acetyl-dehydro-phenylalanine, recently, we studied the positioning of the two phosphito units on the calix[4]arene platform and its effect on the transfer of chirality from the ligand to the substrate (Equation (2) in Figure 2). The two phosphito units were grafted either on distal (**B**) and proximal (**D**) aromatics of the lower rim or on distal (**E**) aromatics of the upper rim. The catalytic system was generated in situ starting from [Rh(cod)_2_]BF_4_ (1 mol%) and the ligand. The runs were performed in CH_2_Cl_2_ at room temperature under 5 bar of hydrogen for 24 h. Under the latter catalytic conditions, the pro-chiral olefin was fully reduced and the enantioselectivities increased in the order **E** (ee 57%) < **D** (ee = 62%) < **B** (ee = 91%). These differences could be explained by steric factors generated by the second coordination sphere of the ligand, i.e., by the calix[4]arene skeleton. In accordance with molecular models, the more sterically constrained active species (encapsulation of the catalytic center inside a molecular pocket), obtained when the diphosphite **B** was employed, led to the more efficient chirality transfer from the ligand to the substrate [18].

Regarding the carbon–carbon bond formation, the most popular reaction is the palladium-catalyzed asymmetric allylic alkylation of 1,3-diphenylprop-2-enyl acetate with dimethyl malonate, released generally with the *N*,*O*-bis(trimethylsilyl)acetamide and acetate salt as bases (Equation (3) in Figure 2). In this context, diphosphine **F**, in which the calix[4]arene platform exhibits an inherent chirality [19], led to a moderate enantiomeric excess of 67% [20]. A higher enantioselectivity (ee = 82%) was reported by Harvey, Jugé, and co-workers using the *C*_2_ symmetry P-chirogenic diphosphine **G**, [Pd(*η*^3^-C_3_H_5_)Cl]_2_ as an organometallic precursor and *^n^*BuLi as the base [21]. The best enantiomeric excess value (ee = 86%) was measured by Manoury and co-workers using the in situ catalytic system resulting from calixarenyl di-ferrocenylphosphine **H** and [Pd(*η*^3^-C_3_H_5_)Cl]_2_ (6 mol%) with MOAc (M = Na or K) as the base. With this system, the enantioselectivity of the product strongly depended on the acetate counterion, and the highest enantiomeric excess value was measured when KOAc was employed [22].

In 2019, our group reported the rhodium-catalyzed asymmetric hydroformylation of styrene (Equation (4) in Figure 2) with diphosphite **E**, in which the two phosphito units were grafted on the upper rim of the calix[4]arene platform. Using the [Rh(acac)(CO)_2_] source of metal (0.1 mol%) and an excess of ligand (5 equiv./Rh) after 24 h at 50 °C in toluene under 20 bar of syngas, the aldehydes were formed in a conversion of 82%, and the 2-phenylpropanal was observed with a b/l ratio of 88:12 and an ee of 89%. Molecular models reveal that the most favorable conformation of the trigonal bipyramidal [RhH(CO)_2_(**E**)] active species has the rhodium atom turned towards the exterior of the cavity with the apical hydride and the equatorial carbon monoxide ligands located in a chiral hindered environment generated by the two binaphthyl moieties [23].

**Figure 4:**
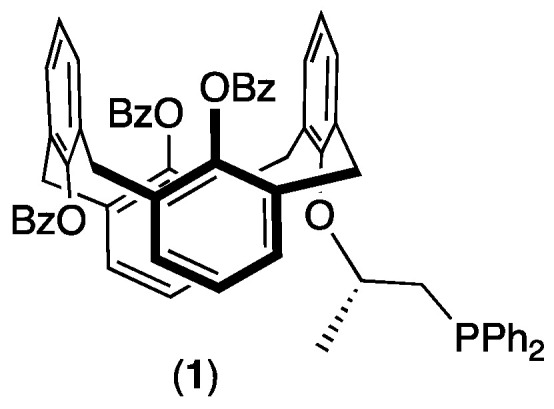
Targeted phosphine **1** build on a calix[4]arene platform.

In this context, and to the best of our knowledge, there is no example of a ruthenium catalyst based on optically calixarenyl phosphine achieving the transfer hydrogenation of ketones. In continuation of our quest to develop a new macrocyclic ligand, herein we report the synthesis of phosphine **1** (Figure 4) and its application in the asymmetric ruthenium-catalyzed reduction of ketones.

## 2. Results and Discussion

### 2.1. Synthesis of the Phosphine Borane Adduct

**Scheme 1:**
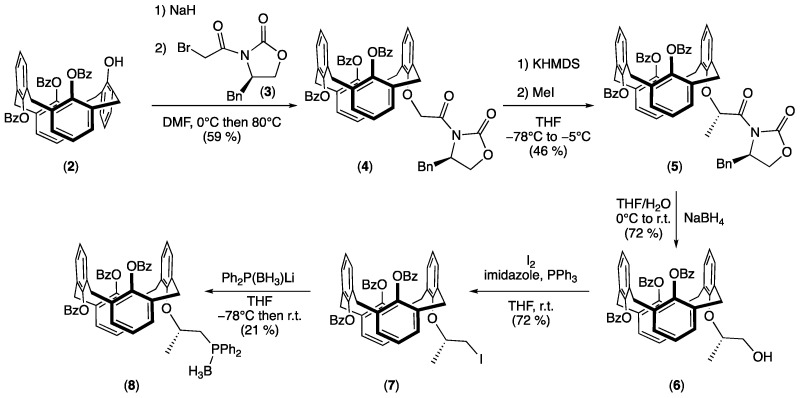
Synthesis of borane adduct **8**.

In continuation of our preliminary research on the optically active calixarenes [24], we reinvestigated our work on Evans’ oxazolidinone calix[4]arene for the synthesis of {25,26,27-tribenzoyl-28-[((*S*)-1-diphenylphosphanyl-propan-2-yl)oxy]-calix[4]arene} borane (**8**) (Scheme 1).

Synthesis of the targeted borane adduct **8** started with the grafting of Evans’ oxazolidinone on the partial cone 25,26,27-tribenzoyl-28-hydroxycalix[4]arene (**2**). Then, a Williamson substitution was carried out in DMF in two steps. First, a deprotonation of the calixarenyl phenol with NaH followed by the addition of (*R*)-4-benzyl-3- (2-bromoacetyl)oxazolidin-2-one (**3**) [25,26]. After purification, the optically pure calixarene **4** was isolated in 59% yield. NMR spectra confirmed the presence of the oxazolidinone substituent. In addition, careful examination of the ^13^C NMR spectrum revealed the presence of two signals at 37.30 and 37.25 ppm for the ArCH_2_Ar methylene carbons. According to the observations of de Mendoza, Prados, and co-workers, such ^13^C NMR values are typical for CH_2_ groups bridging anti-oriented phenol rings [27], which implies a 1,3-alternate conformation of the calix[4]arene skeleton.

**Scheme 2:**
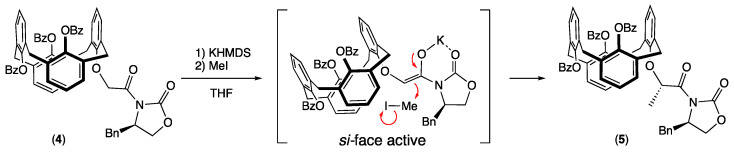
Stereoselective electrophilic addition.

Having in hand the optically pure calixarenyl oxazolidinone **4**, asymmetric methylation, induced by the chiral auxiliary, was achieved on the methylenic position using potassium bis(trimethylsilyl)amide (KHMDS) as the base and methyl iodide as the electrophile [28,29]. The methylated calixarene **5** was isolated in 46% yield as a unique diastereoisomer, as shown by its ^1^H NMR spectrum, where only one signal could be observed at 1.62 ppm (doublet with ^3^*J*_HH_ = 6.5 Hz) for the grafted methyl group. This may be explained by the coordination of the potassium cation between the enolate intermediate and the carbonyl group of the carbamate function. This flat conformation induced a more accessible approach for steric reasons of the electrophile (MeI) on the opposite side (*si*-face) of the benzyl group (Scheme 2) [30].

In agreement with the work of Prashad and co-workers [31], after treatment of Evans’ compound **5** with NaBH_4_ in THF/H_2_O, oxazolidinone-free calixarene **6** was isolated in 72% yield. Its ^13^C NMR spectrum displayed a singlet at 67.47 ppm attributed to the CH_2_OH group resulting from the cleavage of the chiral auxiliary. Iodide derivative **7** was then obtained by reacting **6** with iodine in the presence of imidazole and triphenylphosphine. After purification, calixarene **7** was isolated in 72% yield. The presence of the iodide atom was confirmed by its ^13^C NMR spectrum, in which the CH_2_I signal was observed at 8.89 ppm.

Finally, borane adduct **8** was prepared by reacting in situ-generated nucleophilic lithium diphenylphosphido borane (Ph_2_P(BH_3_)Li) and electrophilic calixarene–iodide **7**. After purification by preparative TLC, air-stable phosphinated compound **8** was isolated in 21% yield. The ^13^C NMR spectrum showed for the CH_2_P moiety a doublet at 31.72 ppm due to phosphorus/carbon coupling (^1^*J*_PC_ = 33.6 Hz). The ^31^P NMR spectrum revealed the presence of a broad singlet at 11.3 ppm close to the reported value for [(2-methoxy)-2-methyl]ethyldiphenylphosphine borane (14.4 pm) [32].

Note that, for the synthetized compounds **3**–**8**, the calix[4]arene skeleton adopted a 1,3-alternate conformation (*vide infra*). Their ^13^C NMR spectra displayed signals in the range of 37.25–38.47 ppm for the ArCH_2_Ar methylene carbons. 

### 2.2. Single-Crystal X-ray Diffraction Study

**Figure 5:**
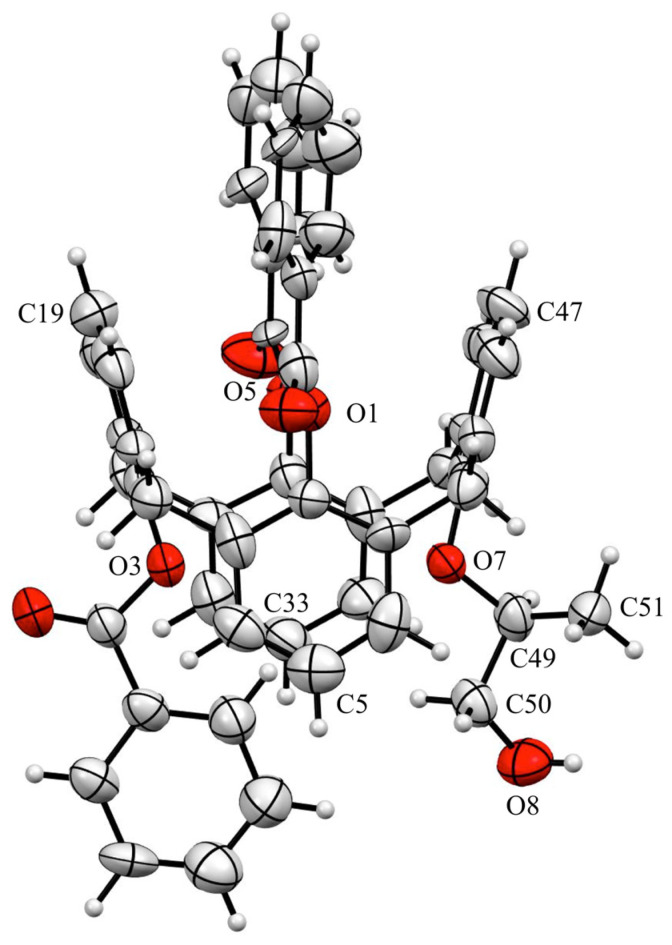
ORTEP drawing of calixarene **6**, 50% probability thermal ellipsoids. For clarity, the molecule of methanol is not represented. Important bond lengths (Å) and angles (°): O7–C49 1.453(12), C49–C51 1.503(15), C49–C50 1.501(14), C50–O8 1.410(13), O7–C49–C51 111.7(9), O7–C49–C50 103.7(7), C51–C49–C50 113.2(10), and C49–C50–O8 112.3(8).

**Figure 6:**
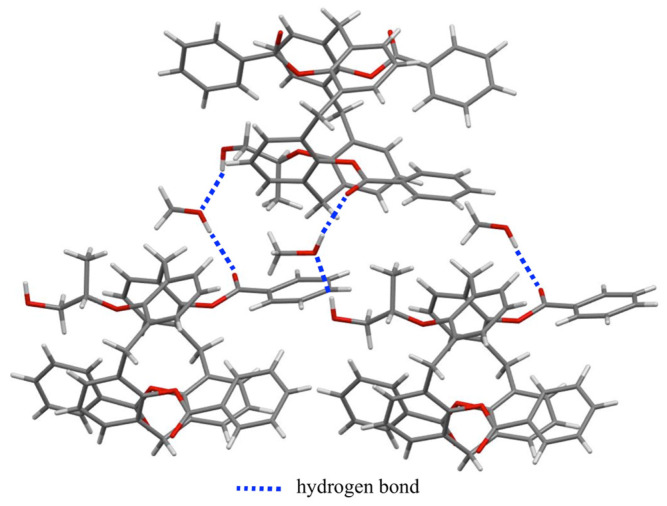
Intermolecular hydrogen bonds.

In 25,26,27-tribenzoyl-28-[((*S*)-1-hydroxypropan-2-yl)oxy]-calix[4]arene (**6**), the conformation of calix[4]arene and the chirality of the stereogenic center of the carbon atom bearing the methyl moiety were confirmed by a single-crystal X-ray diffraction study (Figure 5). Molecule **6** crystallized in the orthorhombic chiral Sohncke space group P2_1_2_1_2_1_ [33]. The unit cell contained four molecules of **6** and four molecules of methanol. Each macrocyclic compound **6** was linked to two molecules of solvent by hydrogen bonds (1.955 Å for the CH(CH_3_)CH_2_OH•••O(H)Me bond and 2.073 Å for the C=O•••HOMe bond corresponding to 2.773(12) and 2.903(12) Å for the O•••O interactions, respectively, as shown in Figure 6). 

This study revealed that the calix[4]arene platform adopted a 1,3-alternate conformation, in which the opposite phenoxy rings displayed dihedral angles of 20.30° and 29.75°, respectively. The separations between the aromatic carbon atoms of opposite phenolic rings were 6.339 (C5–C33) and 6.513 Å (C19–C47). Furthermore, the X-ray diffraction study unambiguously confirmed the (*S*)-configuration of the methylated carbon atom (C49; Flack parameter of 0.03(13) [34]).

Interestingly, careful examination of the structure revealed that the benzoyl aromatic ring attached to the oxygen atom O3 was oriented towards the macrocycle cavity and was located on the bisector of the angle formed by the two phenolic planes, with dihedral angles of 10.67° and 11.43°, respectively. Similarly, the two aromatic cycles of the benzoyl groups attached to the oxygen atoms O1 and O5 were embedded into the calixarenyl cavity with a dihedral angle of 39.51°.

### 2.3. Synthesis of the Ruthenium Complex and Its Catalytic Activity

**Scheme 3:**
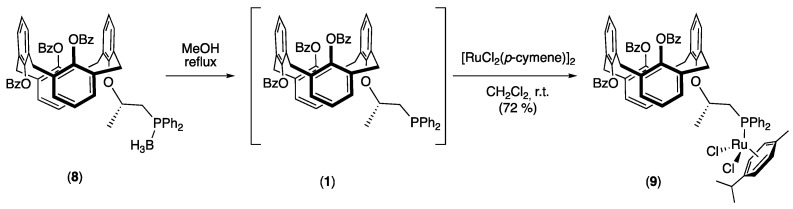
Synthesis of ruthenium complex **9**.

Finally, ruthenium complex **9** was synthetized in two steps from borane adduct **8** without isolation of P(III) intermediate **1** (Scheme 3). First, protected calixarenyl phosphine borane **8** was refluxed in methanol [35], and the reaction was monitored by TLC until full conversion, which was observed after 7 h. The formation of deprotected phosphine **8** was confirmed by ^31^P NMR analysis carried out on the crude reaction mixture, which showed a shift of the unique signal from 11.3 ppm for **8** to −22.2 pm for **1**, in agreement with the value reported for the 1-diphenylphosphinopropan-2-yl acetate (−22 ppm) [36]. The ligand (1 equivalent) was then reacted with the dimeric [RuCl_2_(*p*-cymene)]_2_ organometallic precursor (0.5 equivalent) in dichloromethane. After recrystallization, ruthenium complex **9** was isolated in 72% yield. Its ^31^P spectrum displayed an important upfield shift from −22.2 ppm in the free ligand to 17.2 ppm for the complex. It is interesting to note that the *p*-cymene ligand showed in the ^13^C NMR spectrum a desymmetrization of its four aromatic CH atoms, which appeared as four signals at 91.25, 89.61, 86.65, and 84.23 ppm. In addition, the two CH_3_ of the isopropyl group were, in the ^1^H NMR spectrum, unusually shielded at 0.71 and 0.90 ppm, compared with the classical chemical shift around 0.90 ppm [37,38]. Finally, high-resolution mass spectrometry (HRMS) showing two peaks corresponding to the [M + Na]^+^ (*m*/*z* = 1291.2774) and to the [M + K]^+^ (*m*/*z* = 1307.2502) cations, with the expected isotopic profiles, unambiguously confirmed the formation of ruthenium complex **9**.

Ruthenium complex **9** was tested for the asymmetric reduction of acetophenone. The runs were performed using classical conditions [39] using NaOH as the base in isopropanol (^i^PrOH) as the solvent and hydrogen source. Three experiments, with 1 mol% of catalyst, were carried out at different temperatures for 1 h (Table 1). At 60 °C, a conversion of 60% with an ee of 12% was observed (Table 1, entry 1). Increasing the temperature to 80 °C slightly modified the catalytic outcome; the conversion rose to 68% with an ee of 14% (Table 1, entry 2). Conversely, repeating the run at 100 °C did not favor the catalytic reaction; the conversion was identical as in the case of 80 °C; however, the ee decreased to 4% (Table 1, entry 3). These observed weak enantiomeric excesses were of the same order of magnitude as those generally measured with monodentate ligands [40]. These results show that even when calix[4]arene is a sterically hindered substituent, its influence on the transfer of chirality from the ligand to the substrate is relatively low.

## 3. Materials and Methods

^1^H, ^13^C{^1^H} and ^31^P{^1^H} NMR spectra were recorded with the Bruker Avance III spectrometer (500 MHz) (Billerica, MA, USA) in CDCl_3_ and were calibrated according to residual protonated solvent (*δ* = 7.26 ppm and 77.16 ppm for ^1^H and ^13^C{^1^H} NMR, respectively). ^31^P{^1^H} NMR spectroscopic data were given relative to external H_3_PO_4_. Optical rotations (${\left[\alpha \right]}_{D}^{20}$) were recorded using a 10 cm quartz cuvette with Polarimeter Model 341 (Perkin–Elmer, Waltham, MA, USA) at a wavelength of 589 nm. 25,26,27-tribenzoyl-28-hydroxycalix[4]arene (partial cone) (**2**) [41] and (*R*)-4-benzyl-3-(2-bromoacetyl)oxazolidin-2-one (**3**) [42] were prepared by following the adapted literature procedures, see details in Supplementary Materials. 

### 3.1. Synthesis of 25,26,27-Tribenzoyl-28-[2-((R)-4-benzyl-2-oxooxazolidin-3-yl)-2-oxoethoxy] calix[4]arene (1,3-alternate) *(**4**)*

Oil free NaH (0.046 g, 1.92 mmol, 1.4 equiv.) was added to a white slurry of 25,26,27-tribenzoyl-28-hydroxycalix[4]arene (partial cone) (**2**) (0.837 g, 1.13 mmol) in dry DMF (10 mL) at 0 °C. After 2.5 h at room temperature, a solution of (*R*)-4-benzyl-3-(2-bromoacetyl)oxazolidin-2-one (**3**) (0.380 g, 1.24 mmol, 1.1 equiv.) in dry DMF (3 mL) was added to the previous green solution. After 45 h at 80 °C, CH_2_Cl_2_ (25 mL) and water (10 mL) were added to the stirred reaction mixture. After decantation, the organic layer was treated with water (8 × 10 mL) and brine (10 mL). The organic solution was dried over Na_2_SO_4_, filtered, and concentrated. The resulting crude product was purified by column chromatography on silica gel (AcOEt/cyclohexane with a gradient of solvent from 1:5 to 1:2) to afford calixarene **4** (0.633 g, 59%) as a white solid. (${\left[\alpha \right]}_{D}^{20}$ = −16.1 (c = 1.07, CH_2_Cl_2_). ^1^H NMR (500 MHz, CDCl_3_): *δ* = 7.87–7.85 (m, 4H, benzoyl aromatic CH), 7.77 (t, 2H, benzoyl aromatic CH, ^3^*J*_HH_ = 7.5 Hz), 7.63 (t, 1H, benzoyl aromatic CH, ^3^*J*_HH_ = 7.0 Hz), 7.57 (t, 4H, benzoyl aromatic CH, ^3^*J*_HH_ = 7.5 Hz), 7.55–7.49 (m, 4H, benzoyl aromatic CH), 7.41 (t, 2H, benzyl aromatic CH, ^3^*J*_HH_ = 7.3 Hz), 7.36–7.31 (m, 3H, benzyl aromatic CH), 7.26–7.23 (m, 2H, calixarenyl aromatic CH), 6.70 (d, 2H, calixarenyl aromatic CH, ^3^*J*_HH_ = 7.5 Hz), 6.67–6.64 (m, 2H, calixarenyl aromatic CH), 6.62 (d, 2H, calixarenyl aromatic CH, ^3^*J*_HH_ = 8.0 Hz), 6.60–6.56 (m, 3H, calixarenyl aromatic CH), 6.49 (t, 1H, calixarenyl aromatic CH, ^3^*J*_HH_ = 7.5 Hz), 5.18 and 5.11 (AB system, 2H, NC(=O)CH_2_, ^2^*J*_HH_ = 17.5 Hz), 4.88–4.83 (m, 1H, NCH), 4.39 (dd, 1H, C(=O)OCH_2_, ^2^*J*_HH_ = 9.0 Hz, ^3^*J*_HH_ = 8.6 Hz), 4.32 (dd, 1H, C(=O)OCH_2_, ^2^*J*_HH_ = 9.0 Hz, ^3^*J*_HH_ = 3.1 Hz), 4.05 and 3.59 (AB system, 2H, ArCH_2_Ar, ^2^*J*_HH_ = 15.0 Hz), 4.04 and 3.60 (AB system, 2H, ArCH_2_Ar, ^2^*J*_HH_ = 15.0 Hz), 3.61 (AB system, 4H, ArCH_2_Ar, ^2^*J*_HH_ < 0.5 Hz), 3.50 (A part of an ABX system, 1H, ^2^*J*_AB_ = 13.5 Hz, ^3^*J*_AX_ = 3.0 Hz, CH_2_Ph), 2.94 (B part of an ABX system, 1H, ^2^*J*_AB_ = 13.5 Hz, ^3^*J*_AX_ = 9.5 Hz, CH_2_Ph); ^13^C{^1^H} NMR (126 MHz, CDCl_3_): *δ* = 167.94 (s, C_quat_ NC(=O)CH_2_), 164.58 (s, C_quat_ *C*(=O)Ph), 164.56 (s, C_quat_ C(=O)Ph), 164.31 (s, C_quat_ C(=O)Ph), 156.28 (s, Ar C_quat_ C-O of calixarene), 153.81 (s, C_quat_ NC(=O)O), 148.39 (s, Ar C_quat_ C-O of calixarene), 148.04 (s, Ar C_quat_ C-O of calixarene), 148.00 (s, Ar C_quat_ C-O of calixarene), 135.06–122.84 (Ar C), 70.62 (s, NC(=O)CH_2_), 67.58 (s, C(=O)OCH_2_), 55.13 (s, NCH), 38.20 (s, CH_2_Ph), 37.30 (s, ArCH_2_Ar), 37.25 (s, ArCH_2_Ar) ppm. Elemental analysis (%) calcd. for C_61_H_47_NO_10_ (954.03): C 76.80, H 4.97, N 1.47 found: C 76.65, H 4.88, N 1.36).

### 3.2. Synthesis of 25,26,27-Tribenzoyl-28-{(S)-1-[((R)-4-benzyl-2-oxooxazolidin-3-yl)- 1-oxopropan-2-yl]oxy}-calix[4]arene) (1,3-alternate) *(**5**)*

Calixarene **4** (422 mg, 0.44 mmol) was dissolved in dry THF (11 mL) and cooled to −78 °C before a dropwise addition of KHMDS (potassium bis(trimethylsilyl)amide 0.5 M in toluene, 2 mL, 2.3 equiv.). The addition of MeI (340 μL, 12.3 equiv.) was performed after a period of stirring at −78 °C of 30 min. The cold bath was removed and the stirring was then continued for 4 h, allowing the temperature to rise to −5 °C. The reaction was stopped by addition of a saturated solution of NH_4_Cl (2 mL), water (8 mL), and aqueous HCl 10% (1 mL) to adjust the pH to 4–5. The resulting solution was diluted with AcOEt (15 mL). After decantation, the organic layer was washed successively with water (10 mL) and brine (4 mL) before being dried over Na_2_SO_4_, filtered, and concentrated. The crude white solid was purified by chromatography on silica gel (AcOEt/cyclohexane 1:2 as eluent) to afford the desired white solid **5** (192 mg, 46%). (${\left[\alpha \right]}_{D}^{20}$ = −46.3 (c = 1.0, CHCl_3_). ^1^H NMR (500 MHz, CDCl_3_): *δ* = 7.91 (d, 2H, benzoyl aromatic CH, ^3^*J*_HH_ = 7.5 Hz), 7.78–7.74 (m, 4H, benzoyl aromatic CH), 7.66 (t, 1H, benzoyl aromatic CH, ^3^*J*_HH_ = 7.2 Hz), 7.59–7.53 (m, 4H, benzoyl aromatic CH), 7.50–7.45 (m, 3H, benzoyl and calixarenyl aromatic CH), 7.40–7.34 (m, 5H, benzoyl, benzyl and calixarenyl aromatic CH), 7.30 (t, 1H, benzyl aromatic CH, ^3^*J*_HH_ = 7.2 Hz), 7.22 (d, 2H, benzyl aromatic CH, ^3^*J*_HH_ = 7.0 Hz), 6.74–6.70 (m, 2H, calixarenyl aromatic CH), 6.67–6.64 (m, 3H, calixarenyl aromatic CH), 6.61–6.56 (m, 4H, calixarenyl aromatic CH), 6.44 (t, 1H, calixarenyl aromatic CH, ^3^*J*_HH_ = 7.5 Hz), 6.35 (q, 1H, NC(=O)CHCH_3_, ^3^*J*_HH_ = 6.5 Hz), 4.57–4.53 (m, 1H, NCH), 4.56 and 3.46 (AB system, 2H, ArCH_2_Ar, ^2^*J*_HH_ = 14.5 Hz), 4.12 (dd, 1H, C(=O)OCH_2_, ^3^*J*_HH_ = 9.5 Hz, ^4^*J*_HH_ = 3.0 Hz), 4.04 (d, 1H, C(=O)OCH_2_, ^3^*J*_HH_ = 9.5 Hz), 4.03 and 3.64 (AB system, 2H, ArCH_2_Ar, ^2^*J*_HH_ = 15.0 Hz), 3.63 and 3.61 (AB system, 4H, ArCH_2_Ar, ^2^*J*_HH_ < 0.5 Hz), 3.26 (A part of an ABX system, 1H, ^2^*J*_AB_ = 13.5 Hz, ^3^*J*_AX_ = 3.0 Hz, CH_2_Ph), 2.82 (B part of an ABX system, 1H, ^2^*J*_AB_ = 13.5 Hz, ^3^*J*_AX_ = 9.2 Hz, CH_2_Ph), 1.62 (d, 3H, NC(=O)CHCH_3_, ^3^*J*_HH_ = 6.5 Hz); ^13^C{^1^H} NMR (126 MHz, CDCl_3_): *δ* = 172.55 (s, C_quat_ N*C*(=O)CH_2_), 164.67 (s, C_quat_ C(=O)Ph), 164.43 (s, C_quat_ C(=O)Ph), 164.13 (s, C_quat_ C(=O)Ph), 154.29 (s, Ar C_quat_ C-O of calixarene), 153.32 (s, C_quat_ NC(=O)O), 148.50 (s, Ar C_quat_ C-O of calixarene), 148.29 (s, Ar C_quat_ C-O of calixarene), 135.31–122.41 (Ar C), 71.52 (s, NC(=O)CHCH_3_), 66.83 (s, NC(=O)CH_2_), 55.17 (s, NCH), 38.07 (s, ArCH_2_Ar), 37.77 (s, CH_2_Ph), 37.31 (s, ArCH_2_Ar), 37.28 (s, ArCH_2_Ar), 18.81 (s, NC(=O)CHCH_3_) ppm. Elemental analysis (%) calcd. for C_62_H_49_NO_10_ (968.05): C 76.92, H 5.10, N 1.45 found: C 76.63, H, 5.10, N 1.46).

### 3.3. Synthesis of 25,26,27-Tribenzoyl-28-[((S)-1-hydroxypropan-2-yl)oxy]-calix[4]arene (1,3-alternate) *(**6**)*

An aqueous (0.3 mL) solution of NaBH_4_ (33 mg, 0.87 mmol, 5.3 equiv.) was slowly added to a THF (2 mL) solution of calixarene **5** (160 mg, 0.16 mmol) at 0 °C. The resulting white heterogeneous reaction mixture was warmed to an ambient temperature during the 5 h stirring. The reaction was quenched by the addition of water (5 mL). The pH was adjusted to 2 with aqueous HCl 10% (0.5 mL) and the resulting solution was diluted with CH_2_Cl_2_ (10 mL). After decantation, the organic layer was washed with water (5 mL), brine (4 mL), dried over Na_2_SO_4_, filtered, and concentrated under vacuum to give a white solid. The crude product was purified by chromatography on silica gel (AcOEt/cyclohexane 1:2 as eluent) to afford white alcohol **6** (94 mg, 72%). (${\left[\alpha \right]}_{D}^{20}$ = +3.1 (c = 1.02, CH_2_Cl_2_). ^1^H NMR (500 MHz, CDCl_3_): *δ* = 7.88 (dd, 2H, benzoyl aromatic CH, ^3^*J*_HH_ = 9.0 Hz, ^4^*J*_HH_ = 1.0 Hz), 7.86 (dd, 2H, benzoyl aromatic CH, ^3^*J*_HH_ = 9.0 Hz, ^4^*J*_HH_ = 1.0 Hz), 7.80–7.77 (m, 2H, benzoyl aromatic CH), 7.68–7.65 (m, 1H, benzoyl aromatic CH), 7.61–7.58 (m, 4H, benzoyl aromatic CH), 7.47–7.40 (m, 4H, benzoyl aromatic CH), 7.33 (dd, 1H, calixarenyl aromatic CH, ^3^*J*_HH_ = 8.5 Hz, ^2^*J*_HH_ = 2.5 Hz), 7.26 (dd, 1H, calixarenyl aromatic CH, ^3^*J*_HH_ = 8.5 Hz, ^4^*J*_HH_ = 2.5 Hz), 6.71–6.67 (m, 4H, calixarenyl aromatic CH), 6.65–6.61 (m, 3H, calixarenyl aromatic CH), 6.60–6.57 (m, 2H, calixarenyl aromatic CH), 6.45 (t, 1H, calixarenyl aromatic CH, ^3^*J*_HH_ = 7.5 Hz), 4.70–4.64 (m, 1H, CH_2_CHCH_3_), 3.91–3.82 (m, 2H, CH_2_CHCH_3_), 3.88 and 3.62 (AB system, 2H, ArCH_2_Ar, ^2^*J*_HH_ = 14.0 Hz), 3.86 and 3.54 (AB system, 2H, ArCH_2_Ar, ^2^*J*_HH_ = 14.0 Hz), 3.60 (AB system, 4H, ArCH_2_Ar, ^2^*J*_HH_ < 0.5 Hz), 1.10 (d, 3H, CHCH_3_, ^3^*J*_HH_ = 7.0 Hz); ^13^C{^1^H} NMR (126 MHz, CDCl_3_): *δ* = 164.57 (s, C_quat_ C(=O)Ph), 164.55 (s, C_quat_ C(=O)Ph), 164.10 (s, C_quat_ C(=O)Ph), 153.52 (s, Ar C_quat_ C-O of calixarene), 148.54 (s, Ar C_quat_ C-O of calixarene), 148.30 (s, Ar C_quat_ C-O of calixarene), 148.28 (s, Ar C_quat_ C-O of calixarene), 135.19–122.34 (Ar C), 74.84 (s, CHCH_3_), 67.47 (s, HOCH_2_), 38.19 (s, ArCH_2_Ar), 37.75 (s, ArCH_2_Ar), 37.29 (s, ArCH_2_Ar), 37.27 (s, ArCH_2_Ar), 15.67 (s, CHCH_3_) ppm. Elemental analysis (%) calcd. for C_52_H_42_O_8_ (794.88): C 78.57, H 5.33 found: C 78.46, H 5.36).

### 3.4. Synthesis of 25,26,27-Tribenzoyl-28-[((S)-1-iodopropan-2-yl)oxy]-calix[4]arene (1,3-alternate) *(**7**)*

A solution of iodine (94 mg, 0.37 mmol, 2.2 equiv.) in dry THF (2 mL) was added at room temperature to a clear solution of calixarene **6** (134 mg, 0.17 mmol), imidazole (46 mg, 0.67 mmol, 4.0 equiv.), and triphenylphosphine (94.8 mg, 0.35 mmol, 2.1 equiv.) in dry THF (4 mL). The clear solution rapidly turned to orange with the formation of a white precipitate. After 5 h at room temperature, the reaction was then stopped by the addition of an aqueous solution of Na_2_S_2_O_3_ (1 M, 1 mL) and water (5 mL). After dilution with AcOEt (10 mL) and decantation, the organic layer was washed with water (5 mL), brine (5 mL), dried over Na_2_SO_4_, filtered, and concentrated. The crude with solid was purified by chromatography on a silica gel (AcOEt/cyclohexane 1:3 as eluent) to afford desired compound **7** (109 mg, 72%). (${\left[\alpha \right]}_{D}^{20}$ = +4.60 (c = 0.76, CH_2_Cl_2_). ^1^H NMR (500 MHz, CDCl_3_): *δ* = 7.83 (d, 4H, benzoyl aromatic CH, ^3^*J*_HH_ = 7.0 Hz), 7.76 (t, 2H, benzoyl aromatic CH, ^3^*J*_HH_ = 7.5 Hz), 7.66 (t, 1H, benzoyl aromatic CH, ^3^*J*_HH_ = 7.5 Hz), 7.57 (d, 2H, benzoyl aromatic CH, ^3^*J*_HH_ = 7.5 Hz), 7.55 (d, 2H, benzoyl aromatic CH, ^3^*J*_HH_ = 8.0 Hz), 7.43 (d, 2H, benzoyl aromatic CH, ^3^*J*_HH_ = 8.0 Hz), 7.36 (t, 2H, benzoyl aromatic CH, ^3^*J*_HH_ = 8.0 Hz), 7.22 (t, 1H, calixarenyl aromatic CH, ^3^*J*_HH_ = 7.7 Hz), 7.21 (t, 1H, calixarenyl aromatic CH, ^3^*J*_HH_ = 7.7 Hz), 6.69–6.63 (m, 6H, calixarenyl aromatic CH), 6.61–6.57 (m, 3H, calixarenyl aromatic CH), 6.47 (t, 1H, calixarenyl aromatic CH, ^3^*J*_HH_ = 7.5 Hz), 4.60–4.54 (m, 1H, ICH_2_CHCH_3_), 3.85 and 3.60 (AB system, 2H, ArCH_2_Ar, ^2^*J*_HH_ = 15.0 Hz), 3.83 and 3.60 (AB system, 2H, ArCH_2_Ar, ^2^*J*_HH_ = 15.0 Hz), 3.61 (AB system, 4H, ArCH_2_Ar, ^2^*J*_HH_ < 0.5 Hz), 3.28–3.19 (m, 2H, ICH_2_CHCH_3_), 1.50 (d, 3H, CHCH_3_, ^3^*J*_HH_ = 6.0 Hz); ^13^C{^1^H} NMR (126 MHz, CDCl_3_): *δ* = 164.56 (s, C_quat_ C(=O)Ph), 164.08 (s, C_quat_ C(=O)Ph), 153.72 (s, Ar C_quat_ C-O of calixarene), 148.39 (s, Ar C_quat_ C-O of calixarene), 148.36 (s, Ar C_quat_ C-O of calixarene), 148.33 (s, Ar C_quat_ C-O of calixarene), 135.32–122.62 (Ar C), 75.57 (s, CHCH_3_), 38.21 (s, ArCH_2_Ar), 38.20 (s, ArCH_2_Ar), 37.32 (s, ArCH_2_Ar), 19.48 (s, CHCH_3_), 8.89 (s, ICH_2_) ppm. Elemental analysis (%) calcd. for C_52_H_41_O_7_I (904.78): C 69.03, H 4.57 found: C 68.93, H 4.64).

### 3.5. Synthesis of {25,26,27-Tribenzoyl-28-[((S)-1-diphenylphosphanyl-propan-2-yl)oxy]-calix[4] arene} borane (1,3-alternate) *(**8**)*

A solution of *^n^*BuLi (1.39 M in hexane, 0.11 mL, 2.2 equiv.) was added to a solution of diphenylphosphine borane (34 mg, 2.4 equiv.) in dry THF (0.5 mL) at −78 °C. The resulting yellow solution was stirred at the same temperature for 0.5 h before being added to a solution of calixarene **7** (64 mg, 0.07 mmol) in dry THF (1 mL) at −78 °C. After 0.5 h at −78 °C, the cold bath was removed and the stirred reaction mixture was warmed to room temperature. After 22 h, the reaction was quenched with water (5 mL). AcOEt (10 mL) was added and the reaction mixture was acidified to pH = 3–4 with an aqueous solution of HCl 10% (0.1 mL). After decantation, the organic layer was washed with water (5 mL), brine (5 mL), dried over Na_2_SO_4_, filtered, and concentrated. The crude product was purified by preparative TLC (AcOEt/cyclohexane 1:10 as eluent) to afford the desired white product **8** (14 mg, 21%). ${\left[\alpha \right]}_{D}^{20}$ = −6.41 (c = 0.53, CH_2_Cl_2_). (^1^H NMR (500 MHz, CDCl_3_): *δ* = 7.83 (dd, 2H, benzoyl aromatic CH, ^3^*J*_HH_ = 7.0 Hz, ^4^*J*_HH_ = 1.5 Hz), 7.80 (dd, 2H, benzoyl aromatic CH, ^3^*J*_HH_ = 7.5 Hz, ^4^*J*_HH_ = 1.0 Hz), 7.78 (t, 1H, benzoyl aromatic CH, ^3^*J*_HH_ = 7.5 Hz), 7.76 (t, 1H, benzoyl aromatic CH, ^3^*J*_HH_ = 7.5 Hz), 7.72–7.69 (m, 2H, benzoyl aromatic CH), 7.58–7.54 (m, 4H, benzoyl aromatic CH), 7.53–7.47 (m, 7H, benzoyl and diphenylphosphanyl aromatic CH), 7.39–7.35 (m, 4H, diphenylphosphanyl aromatic CH), 7.31 (dd, 1H, calixarenyl aromatic CH, ^3^*J*_HH_ = 7.5 Hz, ^4^*J*_HH_ = 1.5 Hz), 7.18 (t, 2H, diphenylphosphanyl aromatic CH, ^3^*J*_HH_ = 7.7 Hz), 7.08 (dd, 1H, calixarenyl aromatic CH, ^3^*J*_HH_ = 7.5 Hz, ^4^*J*_HH_ = 1.5 Hz), 6.68–6.65 (m, 3H, calixarenyl aromatic CH), 6.63–6.58 (m, 3H, calixarenyl aromatic CH), 6.56–6.53 (m, 3H, calixarenyl aromatic CH), 6.47 (t, 1H, calixarenyl aromatic CH, ^3^*J*_HH_ = 7.5 Hz), 4.68–4.63 (m, 1H, PCH_2_CHCH_3_), 3.81 and 3.61 (AB system, 2H, ArCH_2_Ar, ^2^*J*_HH_ = 15.0 Hz), 3.59 and 3.58 (AB system, 4H, ArCH_2_Ar, ^2^*J*_HH_ < 0.5 Hz), 3.35 (AB system, 2H, ArCH_2_Ar, ^2^*J*_HH_ < 0.5 Hz), 2.69–2.63 (m, 1H, PCH_2_CHCH_3_), 2.53–2.47 (m, 1H, PCH_2_CHCH_3_), 1.43–0.90 (br signal, 3H, BH_3_), 1.31 (d, 3H, CHCH_3_, ^3^*J*_HH_ = 5.5 Hz); ^13^C{^1^H} NMR (126 MHz, CDCl_3_): *δ* = 164.55 (s, C_quat_ C(=O)Ph), 164.52 (s, C_quat_ C(=O)Ph), 154.05 (s, C_quat_ C(=O)Ph), 153.03 (s, Ar C_quat_ C-O of calixarene), 148.35 (s, Ar C_quat_ C-O of calixarene), 148.31 (s, Ar C_quat_ C-O of calixarene), 148.26 (s, Ar C_quat_ C-O of calixarene), 134.99–122.54 (Ar C), 70.89 (d, PCH_2_CHCH_3_, ^2^*J*_PC_ = 8.7 Hz), 38.47 (s, ArCH_2_Ar), 37.82 (s, ArCH_2_Ar), 37.30 (s, ArCH_2_Ar), 37.28 (s, ArCH_2_Ar), 31.72 (d, PCH_2_, ^1^*J*_PC_ = 33.6 Hz), 21.86 (s, CHCH_3_); ^31^P{^1^H} NMR (121 MHz, CDCl_3_): *δ* = 11.3 (br s, P(BH_3_)Ph_2_) ppm. Elemental analysis (%) calcd. for C_64_H_54_BO_7_P•H_2_O (994.91): C 77.26, H 5.67 found: C 76.98, H 5.52).

### 3.6. Synthesis of Dichloro-P-{25,26,27-tribenzoyl-28-[((S)-1-diphenylphosphanyl-propan-2-yl) oxy]-calix[4]arene} (p-cymene)ruthenium(II) (1,3-alternate) *(**9**)*

In a first step, degassed methanol (3 mL) was added to calixarene **8** (14.0 mg, 14.3 μmol). The suspension was heated at 85 °C. During the heating, the reaction mixture became a clear solution and the reaction was monitored by TLC until full conversion. After 7 h, ^31^P{^1^H} NMR (121 MHz, C_6_D_6_; *δ* = −22.2 pm, PPh_2_) confirmed the formation of P(III) intermediate **1**. The solvent was then removed under reduced pressure and the resulting solid was heated at 50 °C under vacuum for 1 h. White solid phosphine **1** (13.8 mg) was obtained with a quantitative yield and was immediately engaged in the second step without any further purification. Phosphine **1** (13.8 mg, 14.3 μmol) and [RuCl_2_(*p*-cymene)]_2_ (4.4 mg, 7.2 μmol) were dissolved in distilled and degassed CH_2_Cl_2_ (1 mL). The red clear solution was stirred at room temperature for 19 h and then the solvent was removed under reduced pressure to achieve a brown/red solid, which was recrystallized in CH_2_Cl_2/_n-hexane. Ruthenium complex **9** was recovered with a yield of 72% (13.1 mg). (${\left[\alpha \right]}_{D}^{20}$ = +1.8 (c = 0.97, CH_2_Cl_2_). ^1^H NMR (500 MHz, CDCl_3_): *δ* = 7.96 (d, 1H, benzoyl aromatic CH, ^3^*J*_HH_ = 7.5 Hz), 7.94 (d, 1H, benzoyl aromatic CH, ^3^*J*_HH_ = 8.5 Hz), 7.84 (d, 2H, benzoyl aromatic CH, ^3^*J*_HH_ = 7.5 Hz), 7.77 (d, 2H, benzoyl aromatic CH, ^3^*J*_HH_ = 7.0 Hz), 7.76–7.72 (m, 2H, benzoyl aromatic CH), 7.56–7.46 (m, 10H, benzoyl and diphenylphosphanyl aromatic CH), 7.41 (t, 1H, diphenylphosphanyl aromatic CH, ^3^*J*_HH_ = 7.5 Hz), 7.35–7.30 (m, 3H, diphenylphosphanyl aromatic CH), 7.26–7.23 (m, 3H, diphenylphosphanyl aromatic CH), 7.09 (d, 1H, calixarenyl aromatic CH, ^3^*J*_HH_ = 7.5 Hz), 6.91 (d, 1H, calixarenyl aromatic CH, ^3^*J*_HH_ = 7.0 Hz), 6.69 (d, 1H, calixarenyl aromatic CH, ^3^*J*_HH_ = 7.0 Hz), 6.64 (d, 1H, calixarenyl aromatic CH, ^3^*J*_HH_ = 7.5 Hz), 6.60 (d, 1H, calixarenyl aromatic CH, ^3^*J*_HH_ = 7.5 Hz), 6.55–6.43 (m, 7H, calixarenyl aromatic CH), 5.24 and 5.03 (AA’BB’ spin system, 2H, Ar H of *p*-cymene, ^3^*J*_HH_ = 6.0 Hz), 5.03 and 4.93 (AA’BB’ spin system, 2H, Ar H of *p*-cymene, ^3^*J*_HH_ = 6.0 Hz), 4.35–4.28 (m, 1H, PCH_2_CHCH_3_), 3.81 and 3.48 (AB system, 2H, ArCH_2_Ar, ^2^*J*_HH_ = 16.0 Hz), 3.57 and 3.48 (AB system, 2H, ArCH_2_Ar, ^2^*J*_HH_ = 16.0 Hz), 3.53 (AB system, 2H, ArCH_2_Ar, ^2^*J*_HH_ < 0.5 Hz), 3.38 and 3.32 (AB system, 2H, ArCH_2_Ar, ^2^*J*_HH_ = 16.0 Hz), 3.17–3.11 (m, 1H, PCH_2_CHCH_3_), 2.87–2.80 m, 1H, PCH_2_CHCH_3_), 2.47 (hept, 1H, CH(CH_3_)_2_, ^3^*J*_HH_ = 7.0 Hz), 1.80 (s, 3H, CH_3_ of *p*-cymene), 0.90 (d, 3H, CH(CH_3_)_2_, ^3^*J*_HH_ = 7.0 Hz), 0.71 (d, 3H, CH(CH_3_)_2_, ^3^*J*_HH_ = 6.5 Hz), 0.62 (d, 3H, CHCH_3_, ^3^*J*_HH_ = 6.0 Hz); ^13^C{^1^H} NMR (126 MHz, CDCl_3_): *δ* = 164.52 (s, C_quat_ C(=O)Ph), 164.19 (s, C_quat_ C(=O)Ph), 153.21 (s, Ar C_quat_ C-O of calixarene), 148.31 (s, Ar C_quat_ C-O of calixarene), 148.19 (s, Ar C_quat_ C-O of calixarene), 148.11 (s, Ar C_quat_ C-O of calixarene), 136.09–122.44 (Ar C), 108.98 (s, Ar C_quat_ of *p*-cymene), 94.28 (s, Ar C_quat_ of *p*-cymene), 91.25 (d, Ar H of *p*-cymene, ^2^*J*_CP_ = 4.9 Hz), 89.61 (s, Ar H of *p*-cymene), 86.65 (d, Ar H of *p*-cymene, ^2^*J*_CP_ = 4.8 Hz), 84.23 (d, Ar H of *p*-cymene, ^2^*J*_CP_ = 4.8 Hz), 71.39 (d, PCH_2_CHCH_3_, ^2^*J*_CP_ = 5.2 Hz), 38.37 (s, ArCH_2_Ar), 37.94 (s, ArCH_2_Ar), 37.27 (s, ArCH_2_Ar), 30.06 (s, CH(CH_3_)_2_), 29.52 (d, PCH_2_, ^1^*J*_CP_ = 24.3 Hz), 22.15 (s, CH(CH_3_)_2_), 21.82 (s, CHCH_3_), 20.95 (s, CH(CH_3_)_2_), 17.45 (s, CH_3_ of *p*-cymene); ^31^P{^1^H} NMR (121 MHz, CDCl_3_): *δ* = 17.2 (s, PPh_2_) ppm. ESI-MS: displays a peaks at *m*/*z* 1291.2774 [M + Na]^+^ (calcd. for C_74_H_65_Cl_2_O_7_PRuNa: 1291.2781) and 1307.2502 [M + K]^+^ (calcd. for C_74_H_65_Cl_2_O_7_PRuK: 1307.2520)).

### 3.7. X-ray Crystal Structure Analysis

Slow diffusion of methanol into a CH_2_Cl_2_ solution of calixarene **6** led to the formation of single crystals, which were suitable for X-ray analysis. Analysis was carried out on a Bruker APEX II DUO Kappa-CCD diffractometer equipped with an Oxford Cryosystem liquid N_2_ device, using Cu-Kα radiation (λ = 1.54178 Å). The crystal detector distance was 40 mm. The cell parameters were determined (APEX3 software [43]) from reflections taken from three sets of six frames, each at 20 s exposure. The structure was solved using the program SHELXT-2018 [44]. The refinement and all further calculations were carried out using SHELXL-2018 [45]. H-atoms were included in calculated positions and treated as riding atoms using SHELXL default parameters. Non-H atoms were refined anisotropically, using weighted full-matrix least-squares on *F*^2^. A semi-empirical absorption correction was applied using SADABS in APEX3 [43]. Data collection and structure refinement details are given in Table 2.

### 3.8. General Procedure for Ruthenium-Catalyzed Reduction of Acetophenone

A 1 mL vial, under inert atmosphere, was filled with NaOH (1.0 mg, 25 μmol), acetophenone (12 μL, 100 μmol), and a solution of ruthenium complex **9** (1.3 mg, 1 μmol) in ^i^PrOH (0.125 mL, 1.63 mmol, around 16 equiv./ketone). The reaction mixture was heated for 1 h. The solution was diluted with 0.2 mL of CH_2_Cl_2_ and passed through a Millipore filter. An aliquot was analyzed by GC with a Chirasil-DEX CB column (25 m × 0.25 mm) (Agilent Technologies, Santa Clara, CA, USA) to determinate the enantiomeric excess. The remaining solution was concentrated under vacuum and the resulting crude solution was analyzed by ^1^H NMR spectroscopy to determinate the conversion.

## 4. Conclusions

We herein describe the synthesis, in six steps, of 25,26,27-tribenzoyl-28- [((*S*)-1-diphenylphosphanyl-propan-2-yl)oxy]-calix[4]arene (**1**), in which the (*S*)-chirality of the carbon atom bearing the methyl substituent was generated via alkylation of Evans’ oxazolidinone auxiliary. In accordance with the ^13^C NMR spectra made on the calixarenyl compounds, and as confirmed by a single-crystal X-ray diffraction study carried out on the intermediate 25,26,27-tribenzoyl-28-[((*S*)-1-hydroxypropan-2-yl)oxy]-ca- lix[4]arene (**6**), the macrocyclic skeleton adopted a 1,3-alternate conformation.

The arene-ruthenium complex, generated from the phosphine precursor and [RuCl_2_(*p*-cymene)]_2_, was tested in the asymmetric reduction of acetophenone. Although sterically congested, the calix[4]arenyl substituent did not allow an effective transfer of chirality from the ligand to the substrate, resulting in a modest enantiomeric excess.

Further work is aimed at exploiting the potential of Evans’ oxazolidinone methodology for the synthesis of chiral phosphinated calixarenes and their application as ligands in the asymmetric reduction of olefins.

## Figures and Tables

**Table 1:** Ruthenium-catalyzed reduction of acetophenone ^1^.

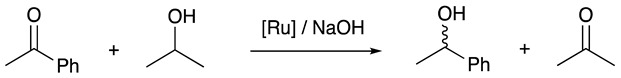			
Entry	Temperature (°C)	Conversion (%)	ee (%)
1	60	60	12
2	80	68	14
3	100	67	4

^1^ Reagents and conditions: ruthenium complex **9** (1 mol%), acetophenone (0.10 mmol), NaOH (0.025 mmol), ^i^PrOH (0.125 mL, 1.63 mmol), 1 h. The conversions were determined on the crude reaction mixture by ^1^H NMR spectroscopy by integrating the CH_3_ signals, and the ees were determined by chiral-phase GC.

**Table 2:** Crystal data and structure refinement parameters for calixarene **6**.

CCDC depository		2313707	color/shape		colorless/block
chemical formula		C_53_H_46_O_9_	formula weight (g mol^−1^)		826.90
crystal system		orthorhombic	space group		P2_1_2_1_2_1_
unit cell parameters	*a* (Å)	12.790(3)	unit cell parameters	*α* (°)	90
	*b* (Å)	12.890(3)		*β* (°)	90
	*c* (Å)	25.477(7)		*γ* (°)	90
volume (Å^3^)		4200.4(17)	Z		4
*D* (g cm^−3^)		1.308	*μ* (mm^−1^)		0.717
*T*_min_, *T*_max_		0.5999, 0.7528	*F*(000)		1744
crystal size (mm)		0.220 × 0.140 × 0.090	index ranges		−15 ≤ *h* ≤ 15
*θ* range for data collection (°)		3.469 ≤ *θ* ≤ 67.329			−13 ≤ *k* ≤ 15
reflections collected		19,727			−27 ≤ *l* ≤ 30
independent/observed		6600/3288	*R*_int_		0.0737
data/restraints/parameters		6600/483/563	goodness-of-fit on *F*^2^		1.045
final *R* indices (*I* > 2.0 *σ*(*I*))		*R*_1_ = 0.0968, w*R*_2_ = 0.2723	*R* indices (all data)		*R*_1_ = 0.2383, w*R*_2_ = 0.3385
Δ*ρ*_max_, Δ*ρ*_min_ (eÅ^−3^)		0.434, −0.687	Flack parameter		0.03(13)

## Data Availability

The data presented in this study are available upon request from the corresponding authors.

## Supplementary Materials

The following supporting information can be downloaded at: https://www.mdpi.com/article/10.3390/molecules29051156/s1. Characterizing data of 25,26,27-tribenzoyl-28-[2-((*R*)-4-benzyl-2-oxooxa- zolidin-3-yl)-2-oxoethoxy]calix[4]arene (1,3-alternate) (**4**) with Figure S1: ^1^H NMR spectrum (CDCl_3_), Figure S2: ^13^C{^1^H} NMR spectrum (CDCl_3_); Characterizing data of 25,26,27-tribenzoyl-28- {(*S*)-1-[((*R*)-4-benzyl-2-oxooxazolidin-3-yl)-1-oxopropan-2-yl]oxy}-calix[4] arene (1,3-alternate) (**5**) with Figure S3: ^1^H NMR spectrum (CDCl_3_), Figure S4: ^13^C{^1^H} NMR spectrum (CDCl_3_); Characterizing data of 25,26,27-tribenzoyl-28-[((*S*)-1-hydroxypropan-2-yl)oxy]-calix[4]arene (1,3-alternate) (**6**) with Figure S5: ^1^H NMR spectrum (CDCl_3_), Figure S6: ^13^C{^1^H} NMR spectrum (CDCl_3_); Characterizing data of 25,26,27-tribenzoyl-28-[((S)-1-iodopropan-2-yl)oxy]-calix[4]arene (1,3-alternate) (**7**) with Figure S7: ^1^H NMR spectrum (CDCl_3_), Figure S8: ^13^C{^1^H} NMR spectrum (CDCl_3_); Characterizing data of {25,26,27-tribenzoyl-28-[((*S*)-1-diphenylphosphanyl-propan-2-yl)oxy]-calix[4]arene} borane (1,3-alternate) (**8**) with Figure S9: ^1^H NMR spectrum (CDCl_3_), Figure S10: ^13^C{^1^H} NMR spectrum (CDCl_3_), Figure S11: ^31^P{^1^H} NMR spectrum (CDCl_3_); Characterizing data of 25,26,27-tribenzoyl-28-[((*S*)-1-diphenylphosphanyl-propan-2-yl)oxy]-calix[4]arene (1,3-alternate) (**1**) with Figure S12: ^31^P{^1^H} NMR spectrum (C_6_D_6_); Characterizing data of dichloro-*P*-{25,26,27- tribenzoyl-28-[((*S*)-1-diphenylphos-phanyl-propan-2-yl)oxy]-calix[4]arene}(*p*-cymene) ruthenium(II) (1,3-alternate) (**9**) with Figure S13: ^1^H NMR spectrum (CDCl_3_), Figure S14: ^13^C{^1^H} NMR spectrum (CDCl_3_), Figure S15: ^31^P{1H} NMR spectrum (CDCl_3_), Figure S16: ^1^H/^1^H COSY spectrum (CDCl_3_), Figure S17: High-resolution mass spectrum (ESI-TOF), Figure S18: High-resolution mass spectrum (ESI-TOF): exp. spectrum (top); calc. spectrum (bottom) for C_74_H_65_Cl_2_O_7_PRuNa, Figure S19: High-resolution mass spectrum (ESI-TOF): exp. spectrum (top); calc. spectrum (bottom) for C_74_H_65_Cl_2_O_7_PRuK.
