# Supplementary material for: Optically Pure Calixarenyl Phosphine via Stereospecific Alkylation on Evans’ Oxazolidinone Moiety
Source: Molecules. 2024 Mar 5;29(5):1156. doi: 10.3390/molecules29051156 (PMC10934826; doi:10.3390/molecules29051156)
Supplement: Supplementary file 1 [file molecules-29-01156-s001.zip › molecules-2873974-supplementary.pdf]

Optically Pure Calixarenyl Phosphine via Stereospecific Alkylation on Evans' Oxazolidinone Moiety

Claude Bauder and David Sémeril

Contents

Characterizing data of 25,26,27-tribenzoyl-28-[2-((*R*)-4-benzyl-2-oxooxazolidin-3-yl)-2-oxoethoxy]calix[4]arene (1,3-alternate) (**4**) p S2

Figure S1. ^{1}H NMR spectrum ($CDCl_3$)

Figure S2. $^{13}C\{^{1}H\}$ NMR spectrum ($CDCl_3$)

Characterizing data of 25,26,27-tribenzoyl-28-{(*S*)-1-[((*R*)-4-benzyl-2-oxooxazolidin-3-yl)-1-oxopropan-2-yl]oxy}-calix[4]arene (1,3-alternate) (**5**) p S4

Figure S3. ^{1}H NMR spectrum ($CDCl_3$)

Figure S4. $^{13}C\{^{1}H\}$ NMR spectrum ($CDCl_3$)

Characterizing data of 25,26,27-tribenzoyl-28-[((*S*)-1-hydroxypropan-2-yl)oxy]-calix[4]arene (1,3-alternate) (**6**) p S6

Figure S5. ^{1}H NMR spectrum ($CDCl_3$)

Figure S6. $^{13}C\{^{1}H\}$ NMR spectrum ($CDCl_3$)

Characterizing data of 25,26,27-tribenzoyl-28-[((*S*)-1-iodopropan-2-yl)oxy]-calix[4]arene (1,3-alternate) (**7**) p S8

Figure S7. ^{1}H NMR spectrum ($CDCl_3$)

Figure S8. $^{13}C\{^{1}H\}$ NMR spectrum ($CDCl_3$)

Characterizing data of {25,26,27-tribenzoyl-28-[((*S*)-1-diphenylphosphanyl-propan-2-yl)oxy]-calix[4]arene} borane (1,3-alternate) (**8**) p S10

Figure S9. ^{1}H NMR spectrum ($CDCl_3$)

Figure S10. $^{13}C\{^{1}H\}$ NMR spectrum ($CDCl_3$)

Figure S11. $^{31}P\{^{1}H\}$ NMR spectrum ($CDCl_3$)

Characterizing data of 25,26,27-tribenzoyl-28-[((*S*)-1-diphenylphosphanyl-propan-2-yl)oxy]-calix[4]arene (1,3-alternate) (**1**) p S12

Figure S12. $^{31}P\{^{1}H\}$ NMR spectrum (C_6D_6)

Characterizing data of dichloro-*P*-{25,26,27-tribenzoyl-28-[((*S*)-1-diphenylphosphanyl-propan-2-yl)oxy]-calix[4]arene}(*p*-cymene)ruthenium(II) (1,3-alternate) (**9**) p S13

Figure S13. ^{1}H NMR spectrum ($CDCl_3$)

Figure S14. $^{13}C\{^{1}H\}$ NMR spectrum ($CDCl_3$)

Figure S15. $^{31}P\{^{1}H\}$ NMR spectrum ($CDCl_3$)

Figure S16. $^{1}H/^{1}H$ COSY spectrum ($CDCl_3$)

Figure S17. High-resolution mass spectrum (ESI-TOF)

Figure S18. High-resolution mass spectrum (ESI-TOF): exp. spectrum (top); calc. spectrum (bottom) for $C_{74}H_{65}Cl_2O_7PRuNa$

Figure S19. High-resolution mass spectrum (ESI-TOF): exp. spectrum (top); calc. spectrum (bottom) for $C_{74}H_{65}Cl_2O_7PRuK$

25,26,27-Tribenzoyl-28-[2-((*R*)-4-benzyl-2-oxooxazolidin-3-yl)-2-oxoethoxy]calix[4]arene (1,3-alternate) (4)

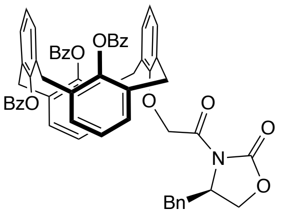

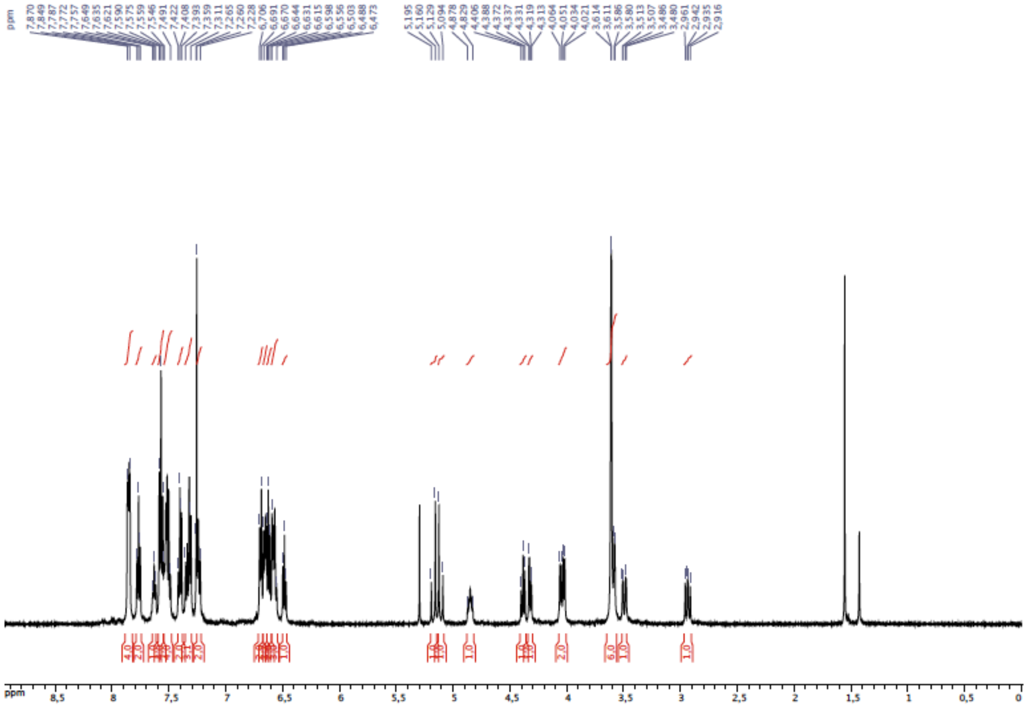

Figure S1. ^{1}H NMR spectrum ($CDCl_3$)

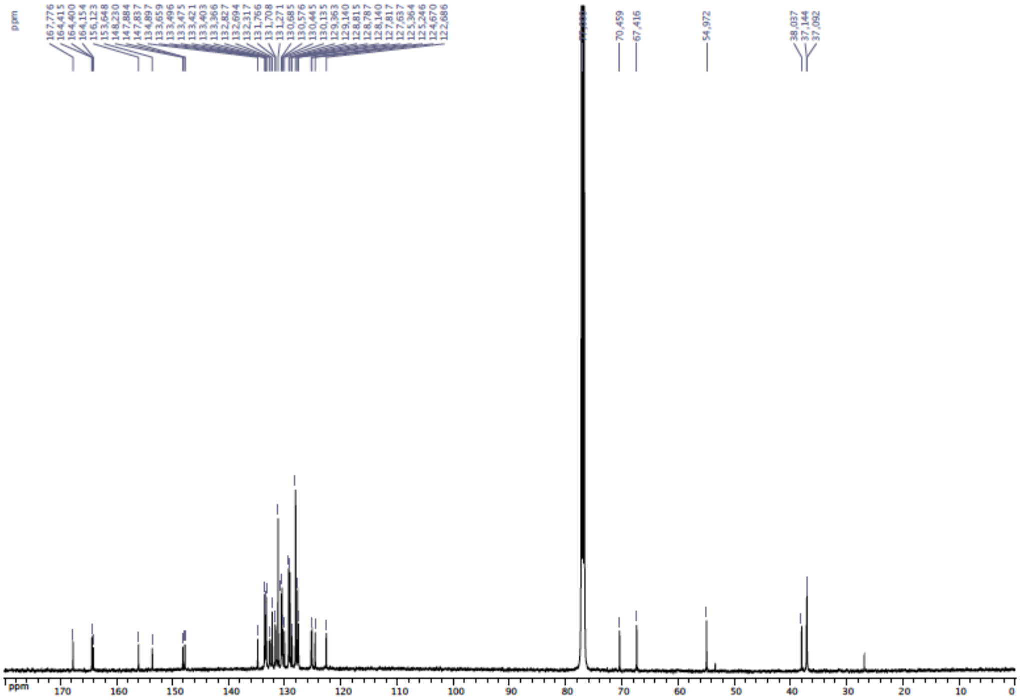

Figure S2. $^{13}C\{^{1}H\}$ NMR spectrum ($CDCl_3$)

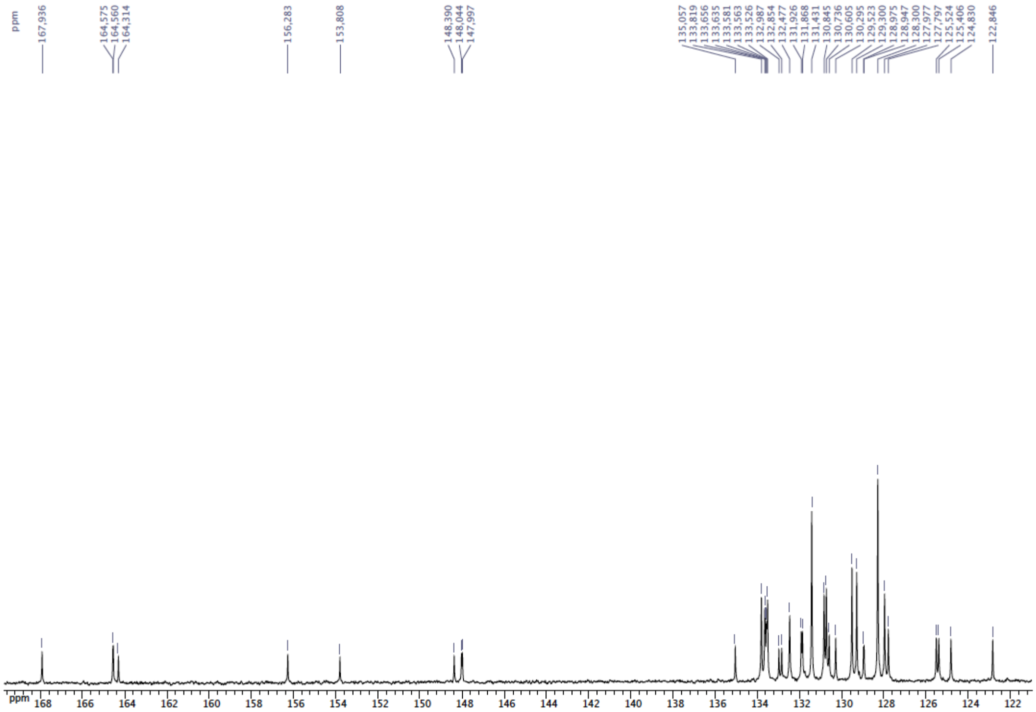

Figure S2-zoom. $^{13}C\{^1H\}$ NMR spectrum ($CDCl_3$)

25,26,27-Tribenzoyl-28-{(*S*)-1-[((*R*)-4-benzyl-2-oxooxazolidin-3-yl)- 1-oxopropan-2-yl]oxy}-calix[4]arene (1,3-alternate) (5)

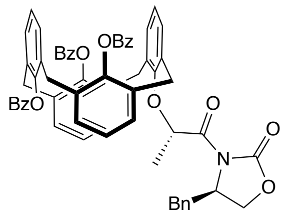

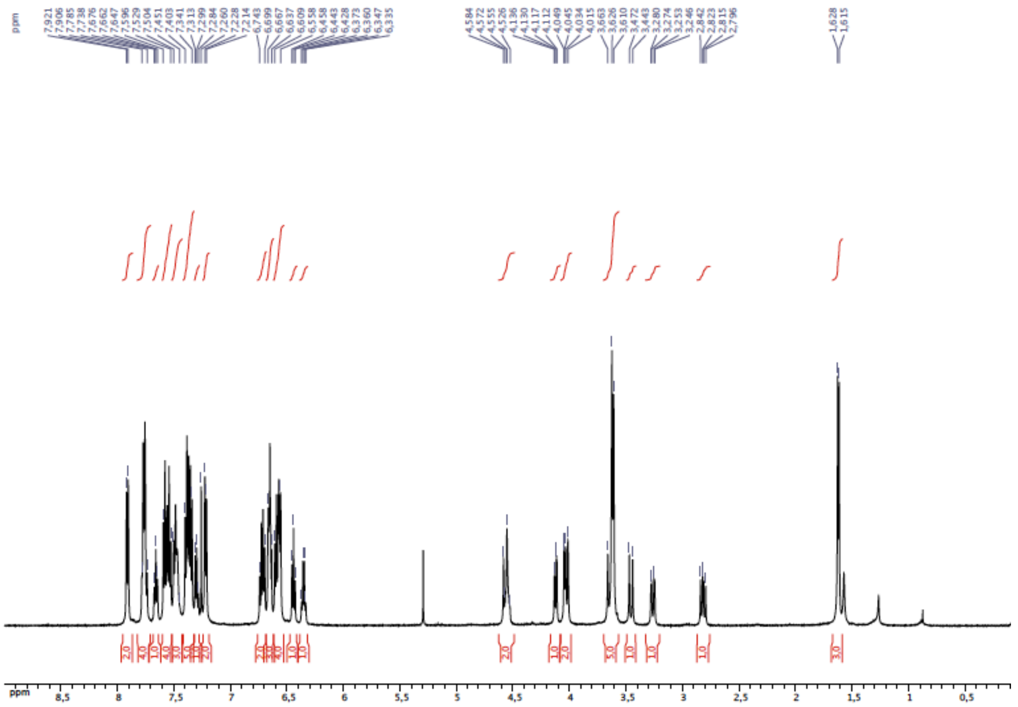

Figure S3. ¹H NMR spectrum ($CDCl_3$)

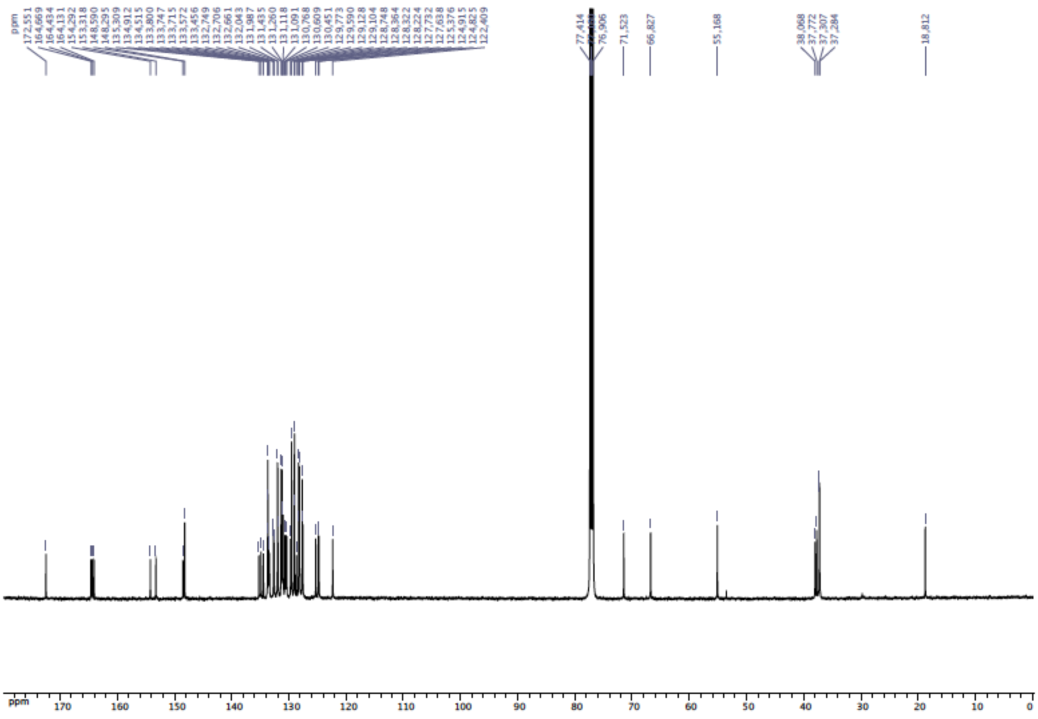

Figure S4. $^{13}C\{^1H\}$ NMR spectrum ($CDCl_3$)

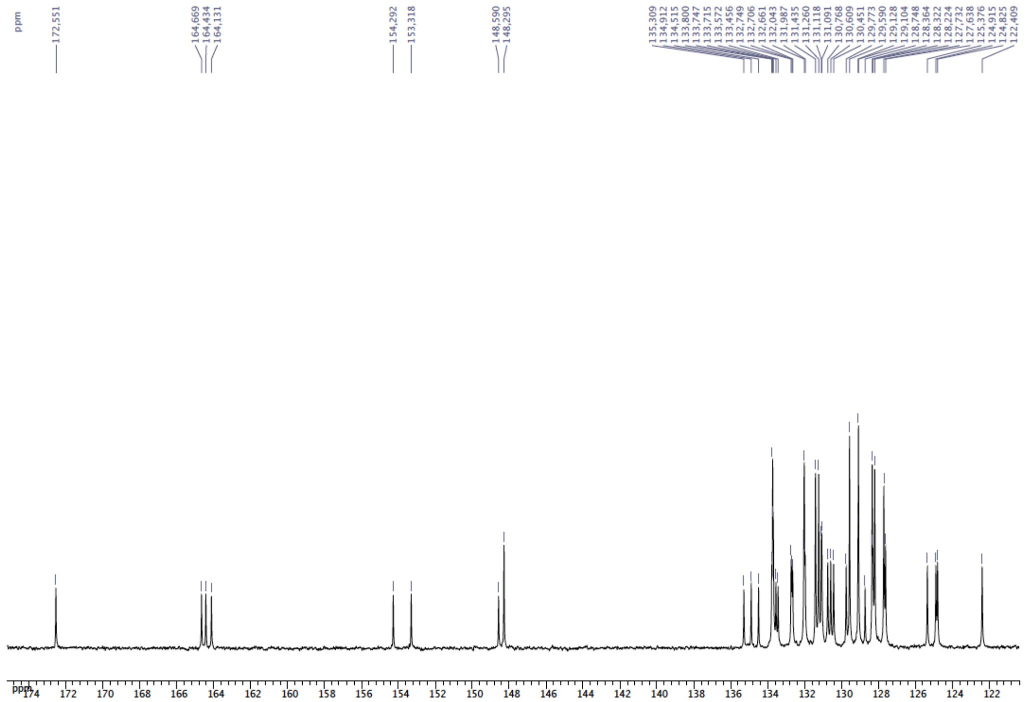

Figure S4-zoom. $^{13}C\{^{1}H\}$ NMR spectrum ($CDCl_3$)

25,26,27-Tribenzoyl-28-[((*S*)-1-hydroxypropan-2-yl)oxy]-calix[4]arene (1,3-alternate) (6)

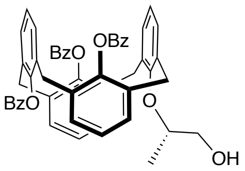

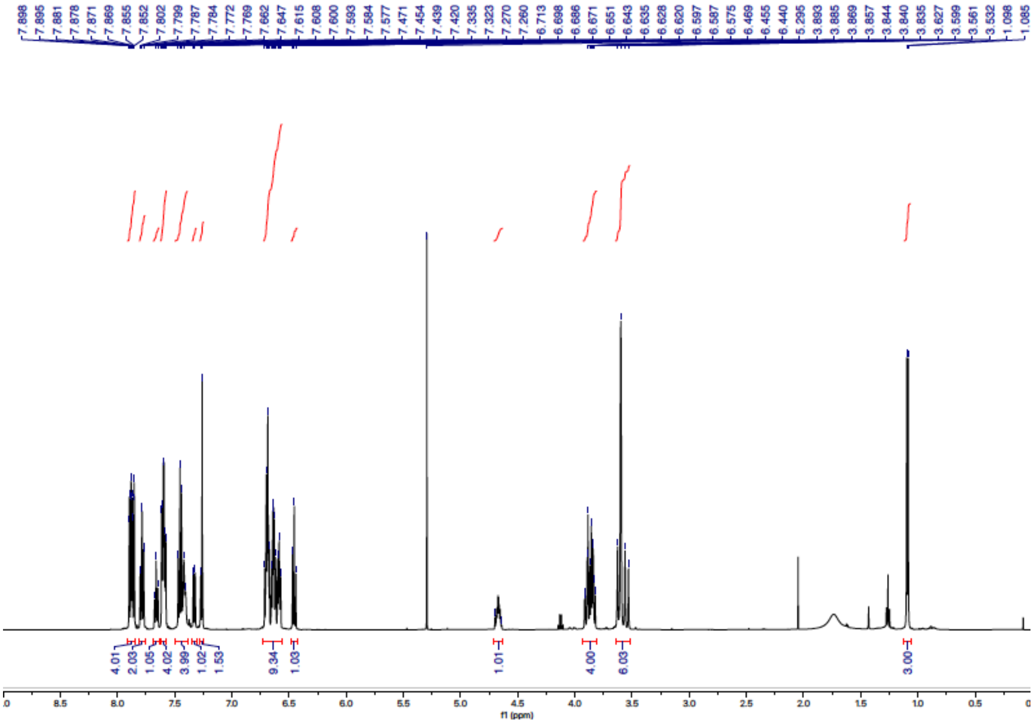

Figure S5. ^{1}H NMR spectrum ($CDCl_3$)

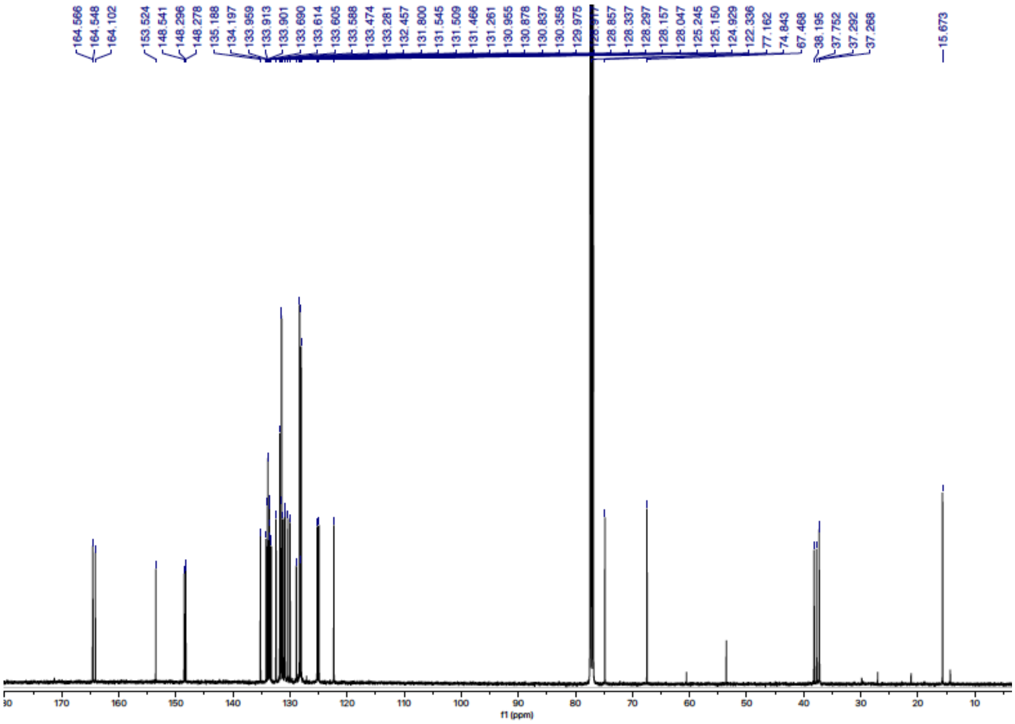

Figure S6. $^{13}C\{^{1}H\}$ NMR spectrum ($CDCl_3$)

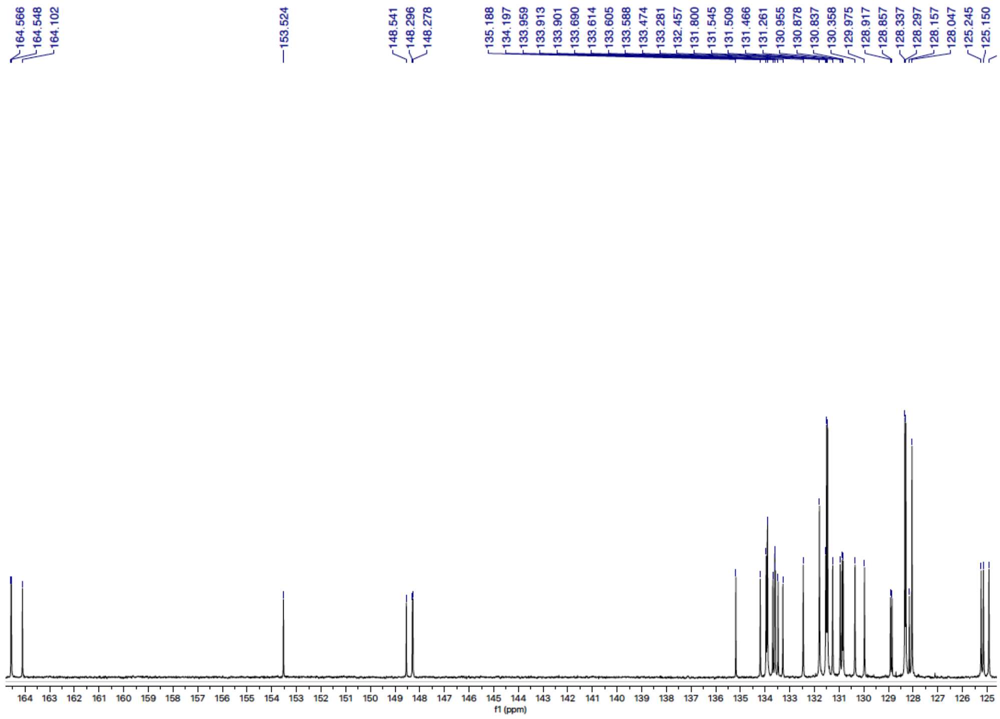

Figure S6-zoom. $^{13}C\{^{1}H\}$ NMR spectrum ($CDCl_3$)

25,26,27-Tribenzoyl-28-[((*S*)-1-iodopropan-2-yl)oxy]-calix[4]arene (1,3-alternate) (7)

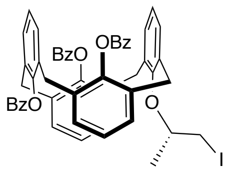

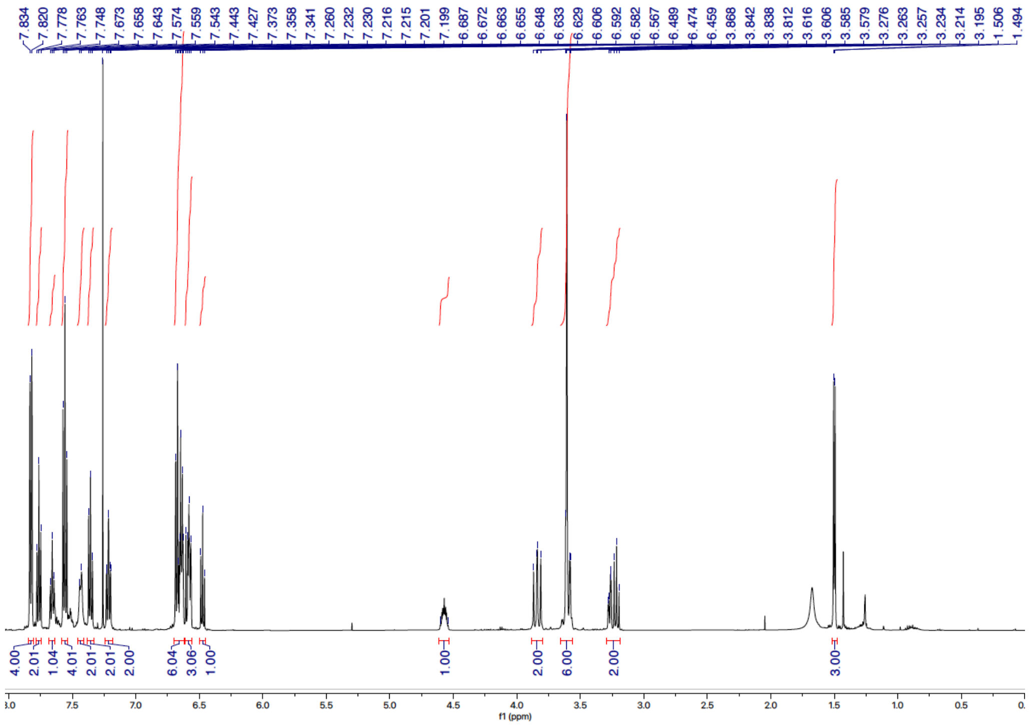

Figure S7. ^{1}H NMR spectrum ($CDCl_3$)

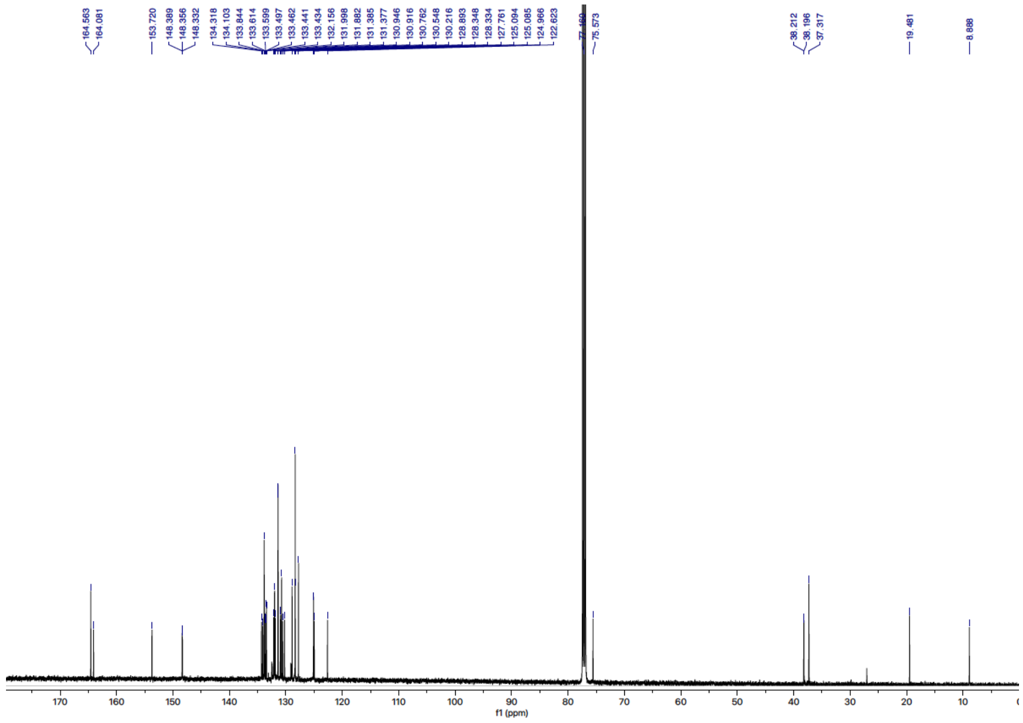

Figure S8. ^{13}C{^{1}H} NMR spectrum ($CDCl_3$)

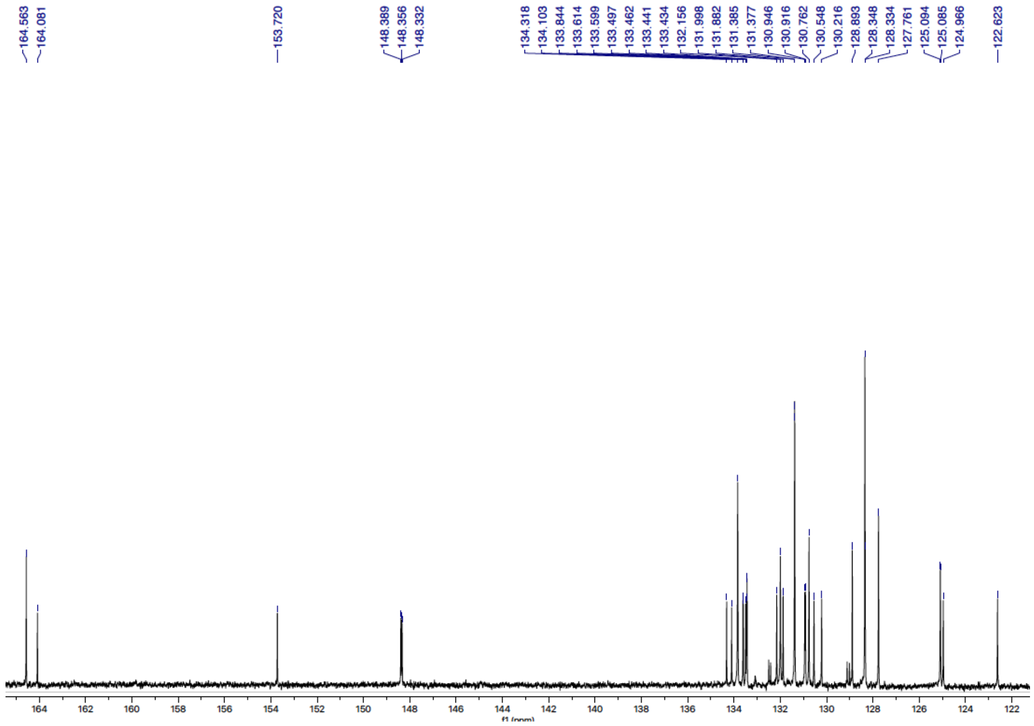

Figure S8-zoom. $^{13}C\{^1H\}$ NMR spectrum ($CDCl_3$)

{25,26,27-Tribenzoyl-28-[((*S*)-1-diphenylphosphanyl-propan-2-yl)oxy]-calix[4]arene} borane (1,3-alternate) (8)

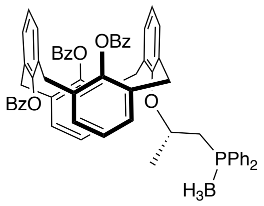

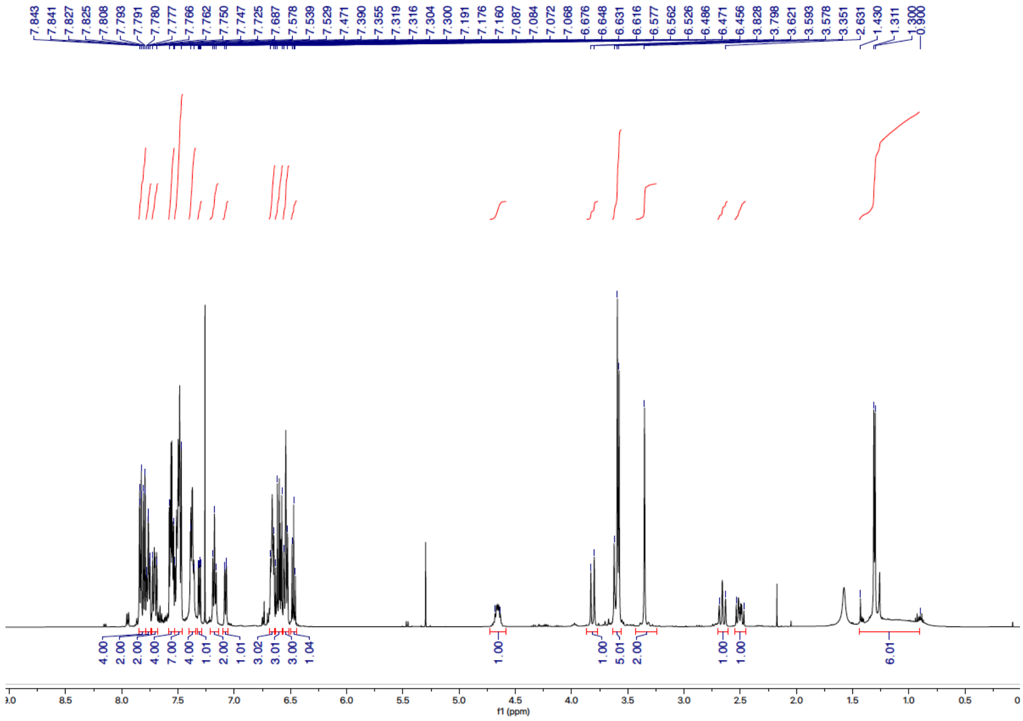

Figure S9. ^{1}H NMR spectrum ($CDCl_3$)

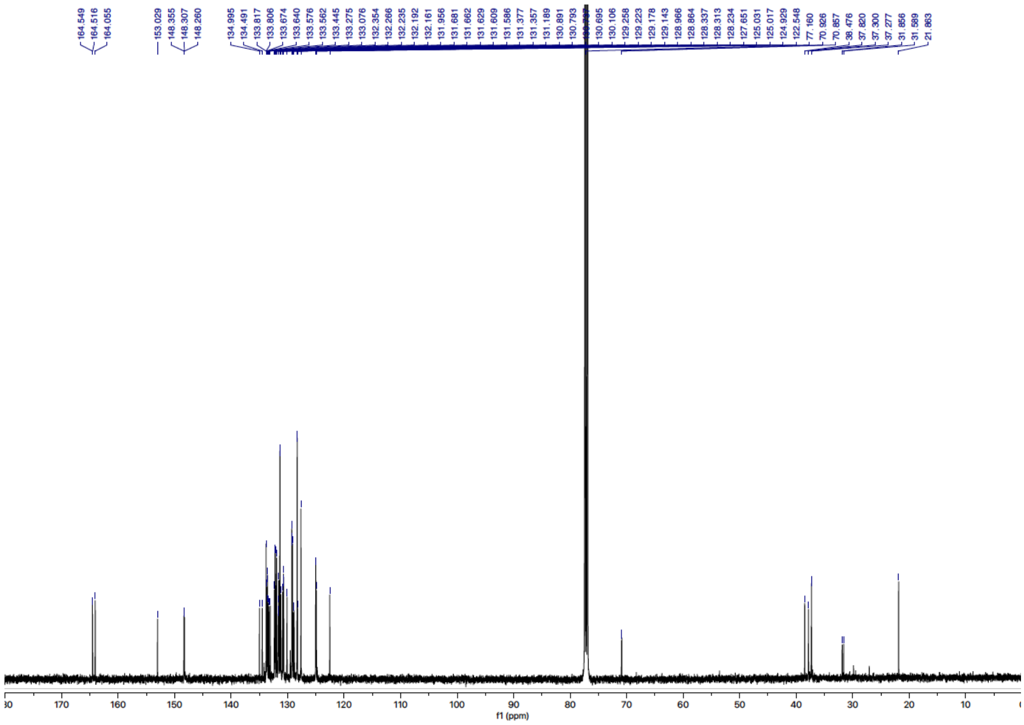

Figure S10. $^{13}C\{^{1}H\}$ NMR spectrum ($CDCl_3$)

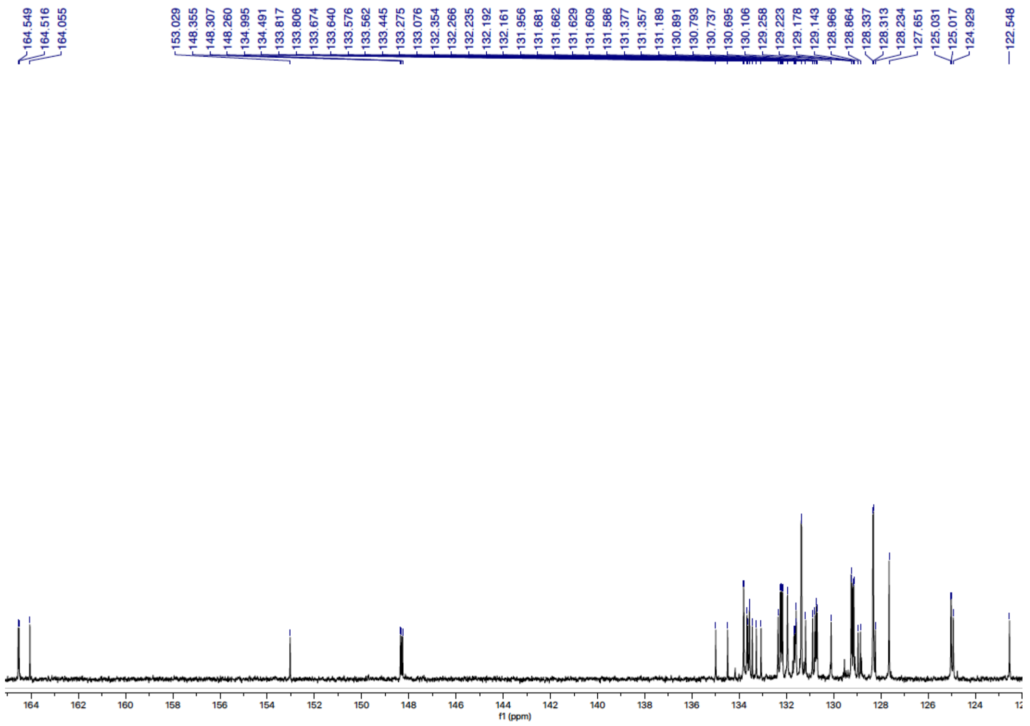

Figure S10-zoom. $^{13}C\{^1H\}$ NMR spectrum ($CDCl_3$)

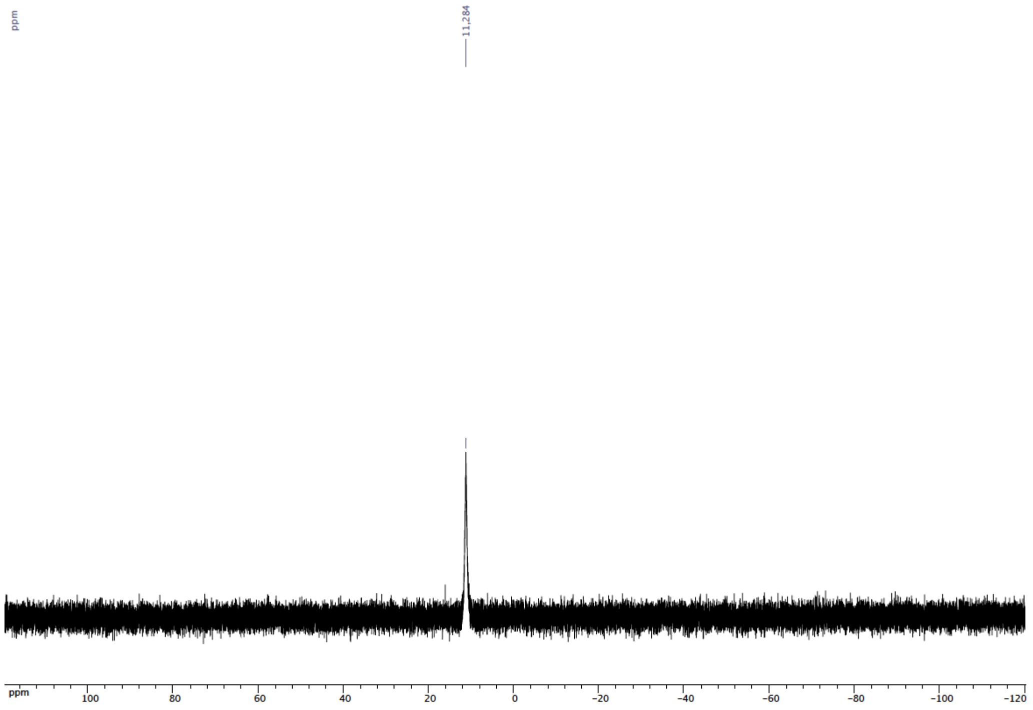

Figure S11. $^{31}P\{^1H\}$ NMR spectrum ($CDCl_3$)

25,26,27-Tribenzoyl-28-[((*S*)-1-diphenylphosphanyl-propan-2-yl)oxy]-calix[4]arene (1,3-alternate) (1)

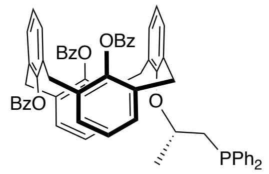

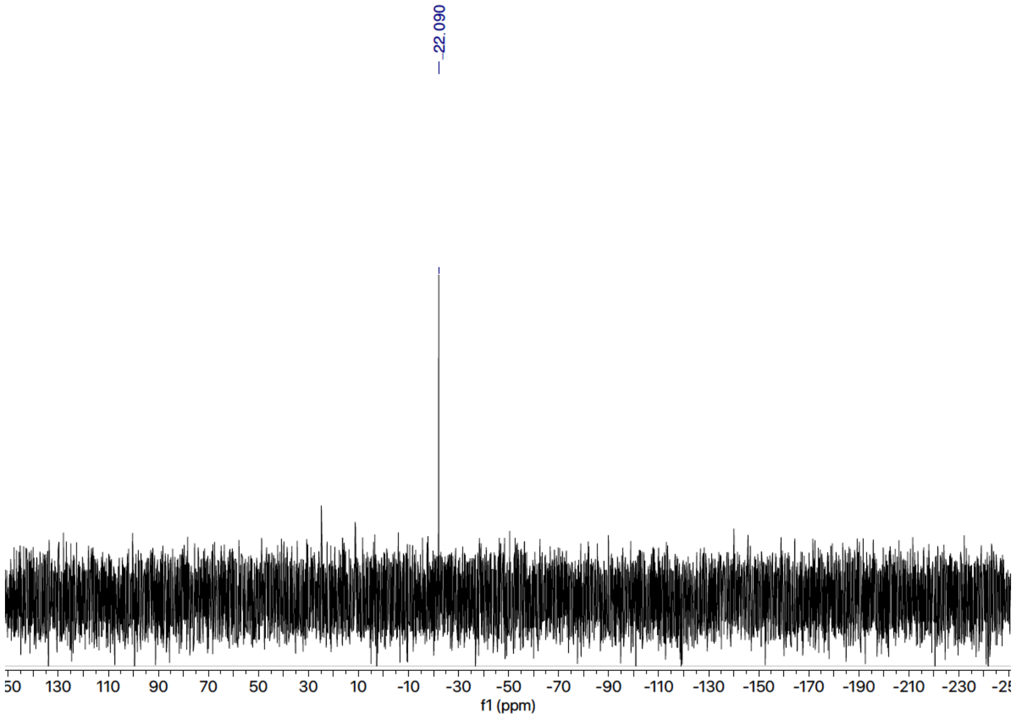

Figure S12. $^{31}P\{^{1}H\}$ NMR spectrum (C_6D_6)

Dichloro-*P*-{25,26,27-tribenzoyl-28-[((*S*)-1-diphenylphosphanyl-propan-2-yl)oxy]-calix[4]arene}(*p*-cymene)ruthenium(II) (1,3-alternate) (9)

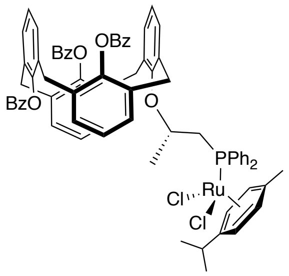

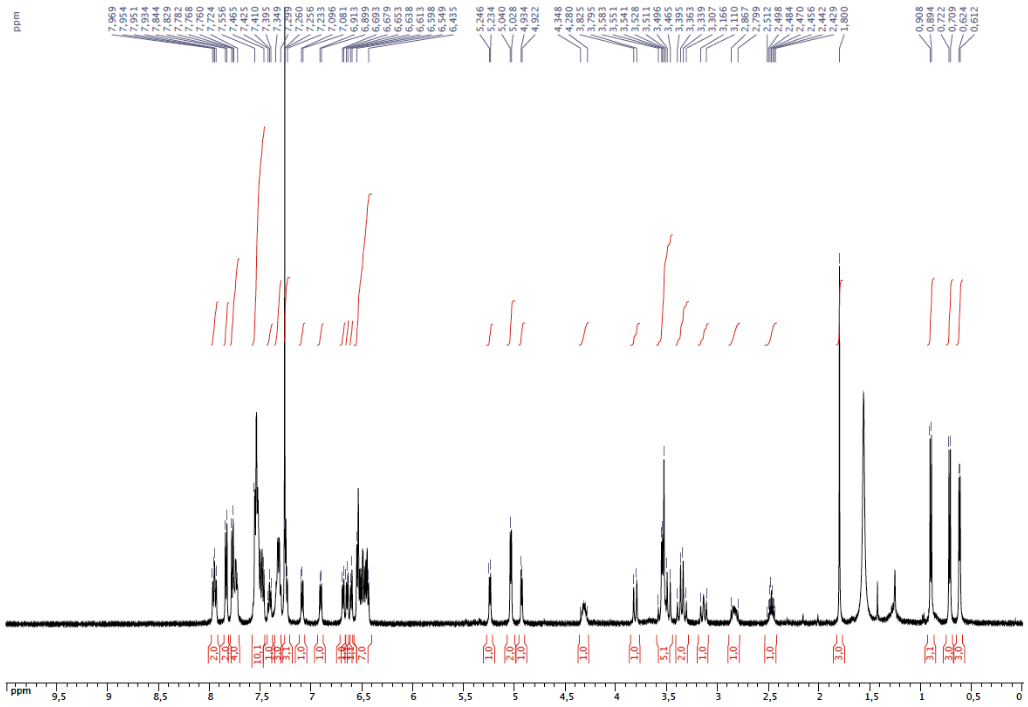

Figure S13. ^{1}H NMR spectrum ($CDCl_3$)

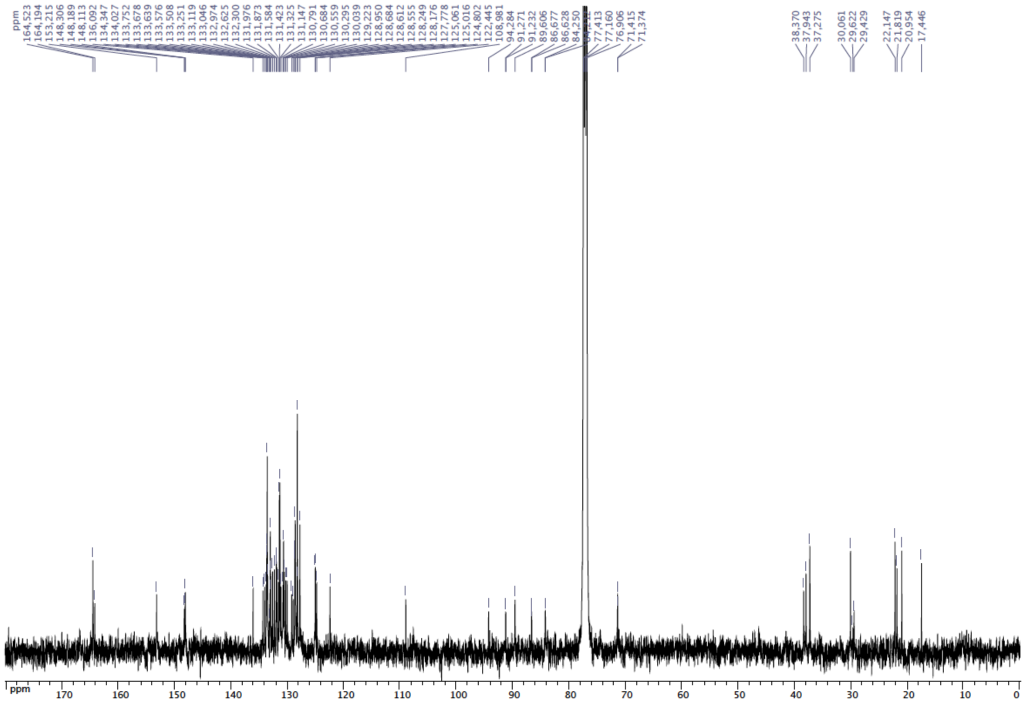

Figure S14. $^{13}C\{^{1}H\}$ NMR spectrum ($CDCl_3$)

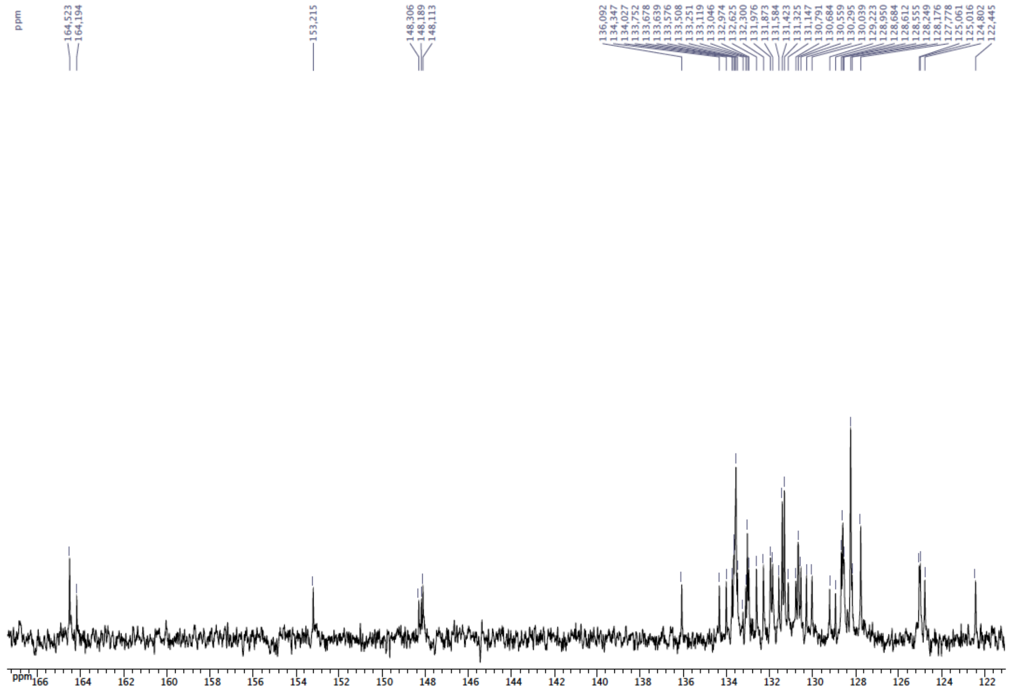

Figure S14-zoom. $^{13}C\{^{1}H\}$ NMR spectrum ($CDCl_3$)

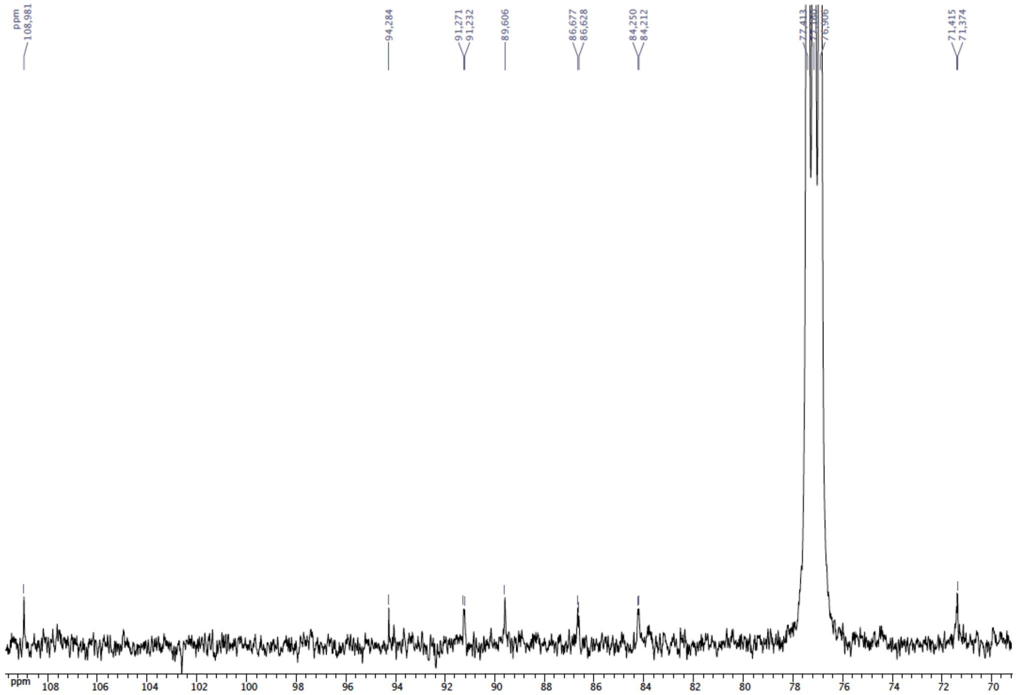

Figure S14-zoom. $^{13}C\{^{1}H\}$ NMR spectrum ($CDCl_3$)

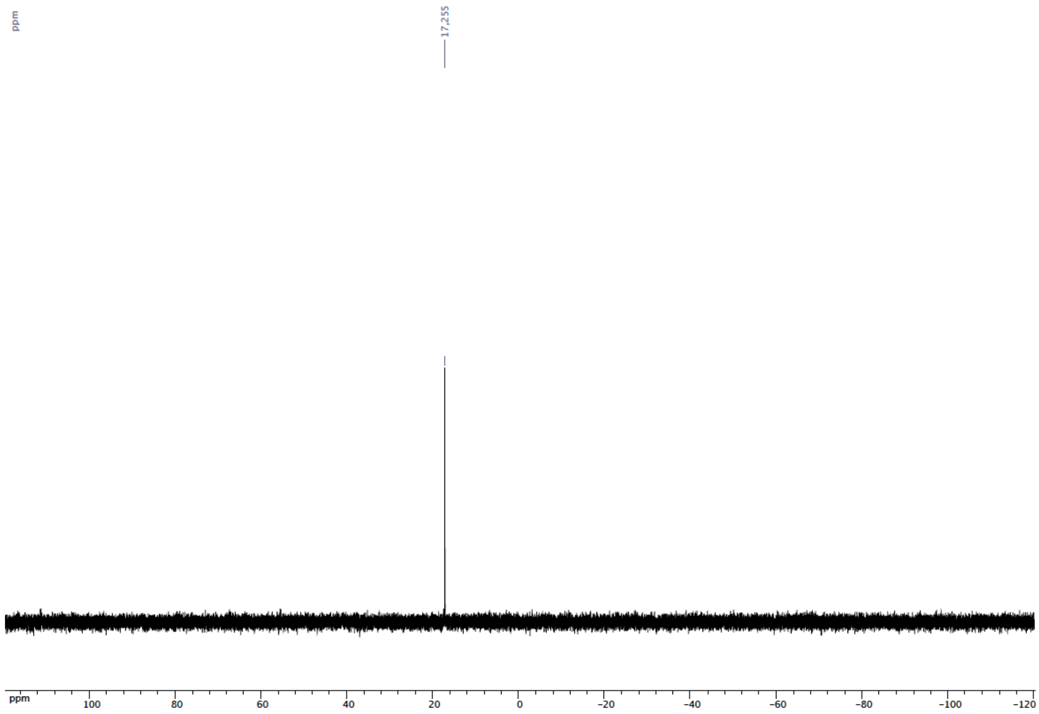

Figure S15. $^{31}P\{^{1}H\}$ NMR spectrum ($CDCl_3$)

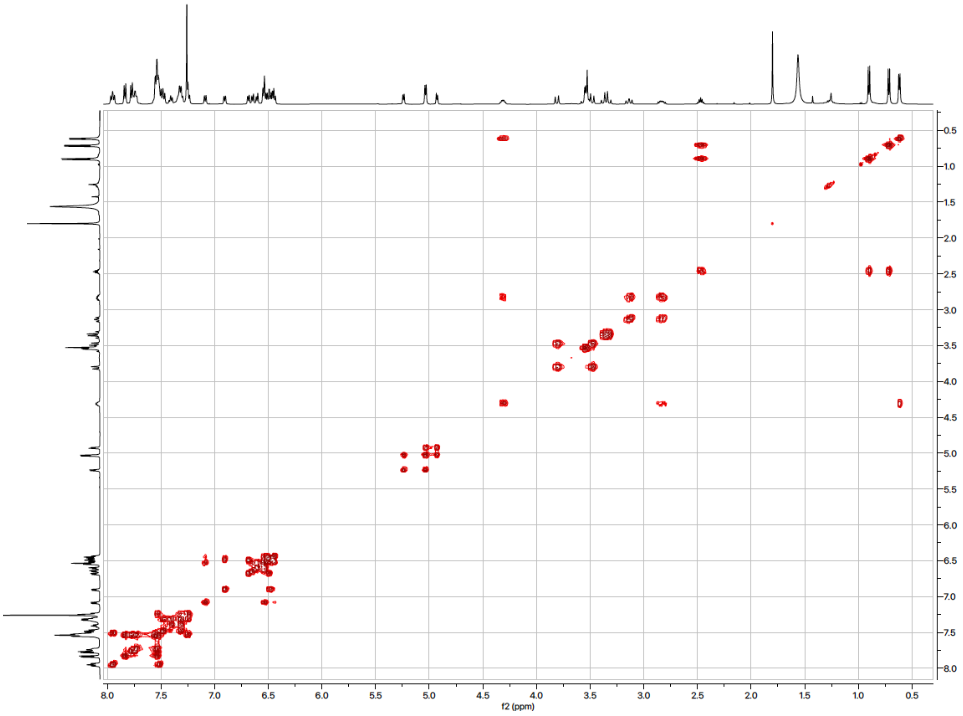

Figure S16. $^{1}H/^{1}H$ COSY spectrum ($CDCl_3$)

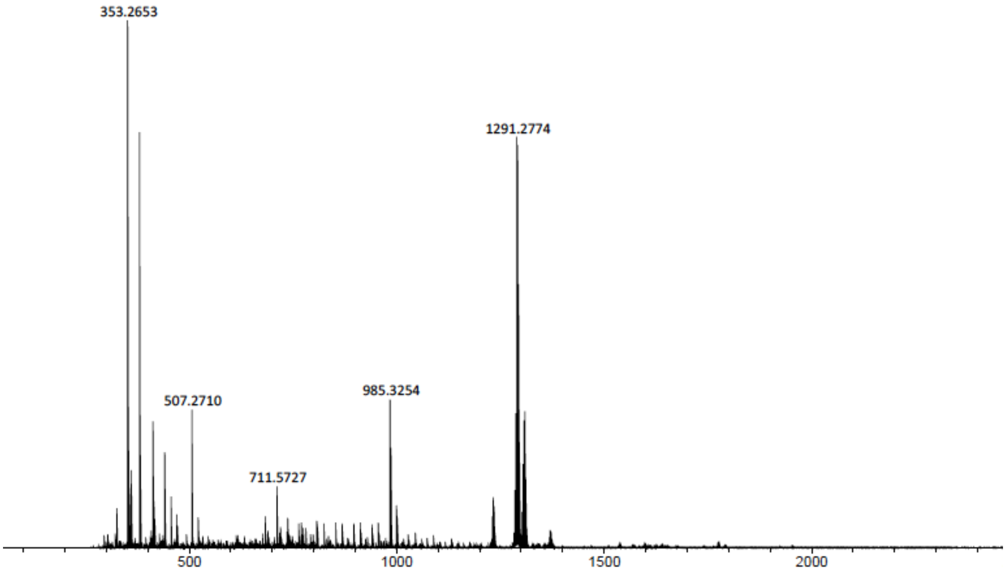

Figure S17. High-resolution mass spectrum (ESI-TOF)

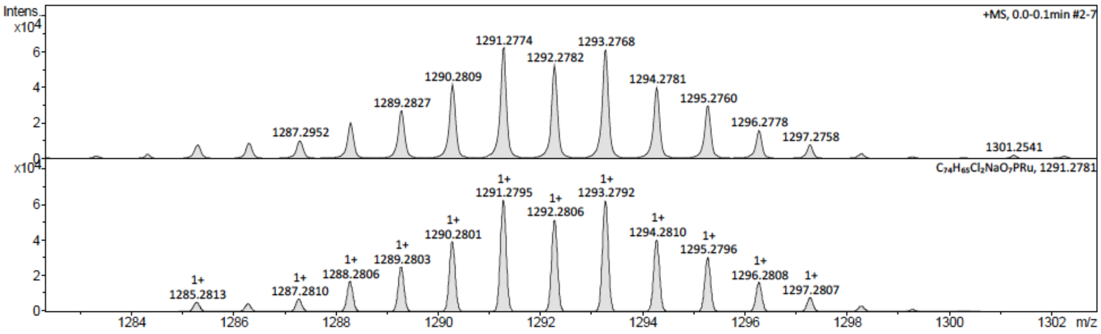

Figure S18. High-resolution mass spectrum (ESI-TOF): exp. spectrum (top); calc. spectrum (bottom) for $C_{74}H_{65}Cl_2O_7PRuNa$

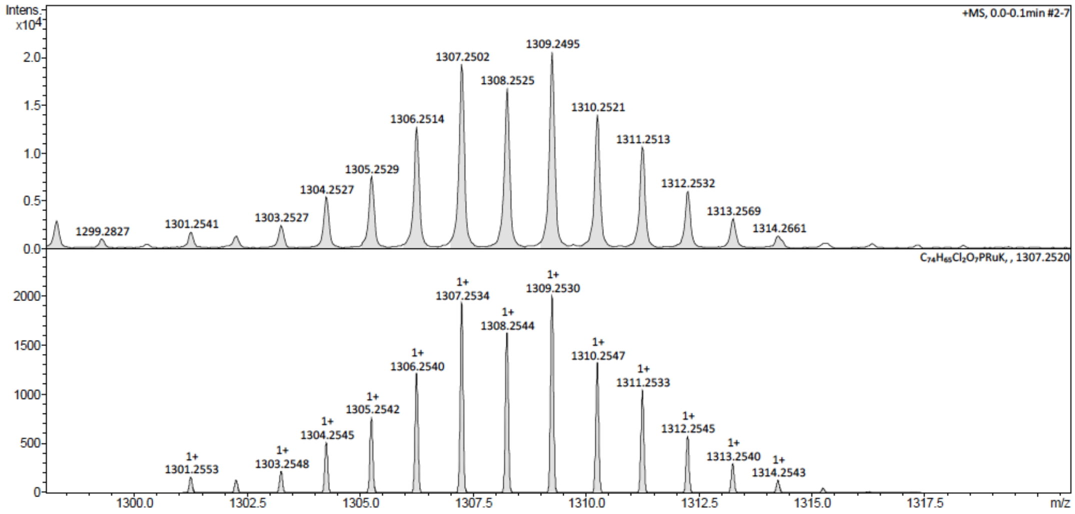

Figure S19. High-resolution mass spectrum (ESI-TOF): exp. spectrum (top); calc. spectrum (bottom) for $C_{74}H_{65}Cl_2O_7PRuK$
